# Local Charge Density Enhancement Strategy in Nitrogen‐rich Covalent Organic Framework for Boosted Iodine Removal From Water

**DOI:** 10.1002/advs.202500697

**Published:** 2025-05-20

**Authors:** Yue Ma, Jinjiao Pan, Huazhen Rong, Lu Liu, Yilei Zhang, Xuewen Cao, Jiacheng Zhang, Tao Liu, Ning Wang, Yihui Yuan

**Affiliations:** ^1^ State Key Laboratory of Marine Resource Utilization in South China Sea Hainan University Haikou 570228 P. R. China

**Keywords:** covalent organic frameworks, iodine removal, local charge density, nitrogen‐rich sites, radioactive iodine

## Abstract

The leakage of nuclear pollution highlights the critical importance of effectively separating radioactive pollutants. Radioactive iodine, a high‐yield fission product of nuclear reactions, poses serious environmental and health risks. However, the lack of efficient adsorbents makes the management of aqueous radioactive iodine pollution a significant challenge. N‐doped materials are among the most recognized adsorbents for iodine removal, but their weak binding affinity and limited number of iodine‐binding N‐sites hinder their practical application. Herein, a covalent organic framework (COFs) named phen‐TPA is synthesized, featuring an increased number and optimized local chemical environment of iodine‐binding N‐sites. This material demonstrates record‐breaking iodine removal kinetics, with a kinetic constant of 14.64 g g^−1^ min^−1^ for aqueous iodine (I_2_), and the highest‐reported iodine adsorption capacity of 11.9 g g^−1^ for aqueous triiodide (I_3_^−^). Large‐scale flow‐through adsorption experiments show that phen‐TPA can remove 99.5% aqueous I_2_ and I_3_^−^ from high‐salinity aqueous environments, highlighting its potential for practical applications.

## Graphical abstract

The covalent organic framework, phen‐TPA, is designed for efficient iodine removal from aqueous solutions, featuring optimized iodine‐binding N‐sites. It shows the fastest iodine removal kinetics for I_2_ and the highest capacity for I_3_^−^. Large‐scale flow‐through tests demonstrate its effective separation of I_2_ and I_3_^−^ in high‐salinity environments, highlighting its potential for practical applications.

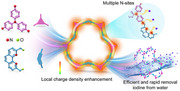

## 1 Introduction

Nuclear power, as a primary global low‐carbon energy source, plays a pivotal role in sustainable development.^[^ ^1^, ^2^ ^]^ However, the growth of the nuclear industry has underscored the critical importance of nuclear waste management and rapid response to nuclear accidents.^[^ ^3^, ^4^, ^5^, ^6^ ^]^ Radioactive iodine, a key fission product of ^235^U, is a major pollutant. Isotopes such as ^129^I (half‐life: 15.7 million years) and ^131^I (half‐life: 8 days) are volatile, highly radioactive, toxic, and capable of disrupting human metabolic processes.^[^ ^7^, ^8^ ^]^ Catastrophic nuclear events, including the Chernobyl and Fukushima disasters, have released large quantities of radioactive iodine into the atmosphere and aquatic systems, contaminating oceans, rivers, and groundwater.^[^ ^9^, ^10^ ^]^ These releases pose severe risks to environmental safety and human health, making the efficient removal of radioactive iodine from water a critical environmental and public health priority.

Solid‐phase adsorption is widely recognized as one of the most effective methods for iodine capture.^[^ ^11^, ^12^, ^13^ ^]^ Traditional inorganic adsorbents, including zeolites, silver‐doped silicon materials, and activated carbon, have been employed for removing radioactive iodine.^[^ ^14^, ^15^, ^16^, ^17^ ^]^ However, these materials often exhibit low efficiency in aqueous environments, limiting their practical applications.^[^ ^18^ ^]^ Recently, porous organic materials such as metal‐organic frameworks (MOFs), porous organic polymers (POPs), and covalent organic frameworks (COFs) have attracted considerable attention for iodine capture.^[^ ^19^, ^20^, ^21^, ^22^, ^23^, ^24^, ^25^ ^]^ Despite their promise, MOFs generally suffer from poor stability during iodine adsorption, while the complex and irregular pore structures of POPs are prone to blockage, hindering their adsorption performance.^[^ ^26^, ^27^ ^]^ In contrast, COFs, a class of crystalline porous materials interconnected via covalent bonds, exhibit exceptional structural stability and uniformly ordered pores.^[^ ^28^, ^29^, ^30^, ^31^, ^32^, ^33^, ^34^, ^35^, ^36^ ^]^ This unique architecture prevents pore occlusion during iodine adsorption, offering significant potential for efficient iodine capture.^[^ ^37^ ^]^ Several COFs have demonstrated remarkably high iodine adsorption capacities.^[^ ^38^, ^39^ ^]^ However, most COFs are optimized for capturing gaseous iodine species, such as I_2_ and methyl iodide, and only a limited number are effective for removing aqueous iodine.^[^ ^40^, ^41^ ^]^ This limitation arises from the insufficient binding affinity of most COFs to aqueous iodine, resulting in slow adsorption kinetics and low adsorption capacity. To address these challenges, rational design of COFs with enhanced binding affinity and optimized pore structures holds great promise for advancing high‐performance materials for aqueous iodine removal. By leveraging their desirable porosity, stability, and modular design, COFs represent a promising frontier in tackling iodine contamination in water systems.

**Figure 1:**
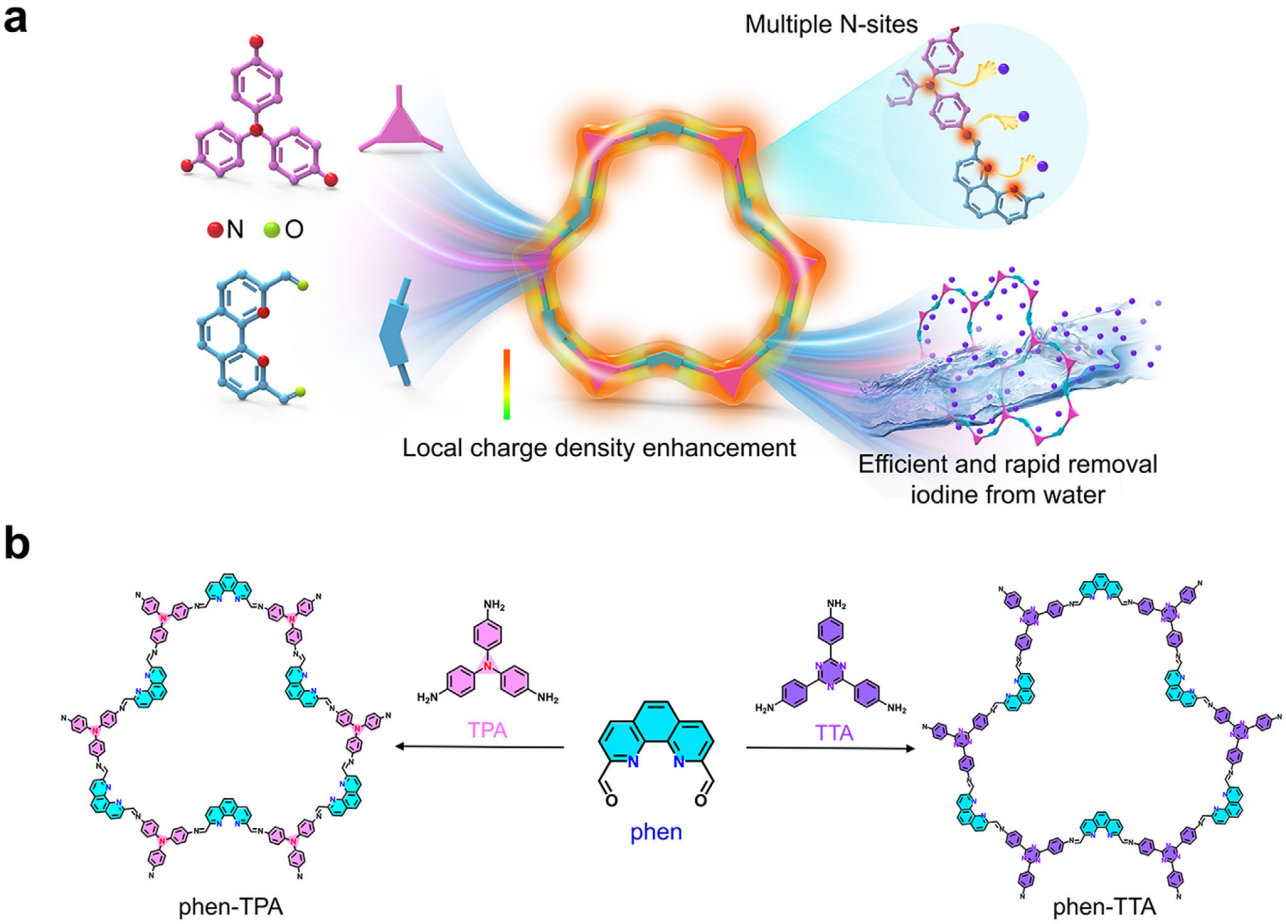
Design of aqueous iodine absorbents with high local charge density and multiple N‐sites. a) A schematic diagram of the phen‐TPA with high local charge density and multiple N‐sites removal iodine from water. b) Synthesis route of phen‐TPA and phen‐TTA.

To date, COFs developed for iodine adsorption primarily rely on the incorporation of heteroatoms, π‐conjugated systems, and ionic functional groups.^[^ ^30^, ^40^ ^]^ Among these, nitrogen‐containing heteroatoms and heterocycles are widely recognized as effective functional groups for iodine binding, due to their strong affinity for iodine. However, existing N‐doped COFs face limitations, including an insufficient number of iodine‐binding N‐sites and inadequately optimized local chemical environments. As a result, the iodine adsorption performance of the current N‐doped COFs remains suboptimal, particularly for addressing aqueous iodine contamination caused by sudden nuclear accidents or radioactive wastewater in the nuclear industry. In this study, two COFs, designated as phen‐TPA and phen‐TTA, were designed with an increased number of iodine‐binding N‐sites to enhance iodine adsorption capacity. Notably, phen‐TPA demonstrated superior performance due to its enhanced molecular‐level local charge density at the iodine‐binding N‐sites, further improving its iodine adsorption capacity and enabling ultrafast adsorption kinetics. This finding underscores the importance of a well‐designed molecular‐level local chemical environment, which is shown to be more impactful than merely increasing the number of binding sites for improving the iodine adsorption performance of COFs (**Figure** **1**a). As a result, phen‐TPA demonstrates record‐breaking iodine removal kinetics with a kinetics constant of 14.64 g g^−1^ min^−1^ for aqueous I_2_ and the best‐performing iodide adsorption capacity of 11.9 g g^−1^ for aqueous I_3_^−^. Large‐scale flow‐through adsorption experiments reveal that phen‐TPA can remove 99.5% aqueous iodine. Furthermore, phen‐TPA can efficiently separate I_2_ and I_3_^−^ from seawater with high salinity, thereby greatly broadening the application of such COF adsorbent. These findings not only propose a promising strategy for the rational chemical design of high‐performance aqueous iodine adsorbents but also offer a viable technical solution for addressing nuclear emergencies and managing radioactive wastewater.

## 2 Results

### 2.1 Synthesis and Structure Characterizations

In this study, two covalent organic frameworks (COFs), phen‐TPA and phen‐TTA, were synthesized as brick‐red and brown powders, respectively, through a polycondensation reaction at 120 °C over 5 days. The reaction involved 1,10‐phenanthroline‐2,9‐diformaldehyde (phen) and tris(4‐aminophenyl)amine (TPA) or tris(4‐aminophenyl)triazine (TTA) (Figure 1b). The as‐synthesised COFs were characterised using Fourier‐transform infrared spectra (FT‐IR) and ^13^C cross‐polarization magic angle spinning (CP/MAS) NMR spectroscopy. The appearance of a new C═N vibration band at 1602 and 1617 cm^−1^ in phen‐TPA and phen‐TTA, respectively, coupled with the disappearance of the C═O vibration band from phen and ‐NH_2_ vibration band from TPA and TTA, confirmed the successful formation of Schiff‐base imine bonds during the reaction (Figure S1, Supporting Information). The ^13^C NMR spectra revealed characteristic imine carbon chemical shifts at 153 ppm and 149 ppm for phen‐TPA and phen‐TTA, respectively, further validating the successful synthesis of the targeted COFs (Figure S2, Supporting Information).^[^ ^42^, ^43^ ^]^

Powder X‐ray diffraction (PXRD) analyses were performed on the phen‐TPA and phen‐TTA to investigate their structure properties. The experimental PXRD patterns of two COFs aligned closely with the calculated patterns for the eclipsed stacking models (Figure S3, Supporting Information). High‐resolution transmission electron microscopy (HR‐TEM) and selected area electron diffraction (SAED) images confirmed the crystalline nature of both COFs, displaying regularly arranged lattice fringes and distinct diffraction spots (Figure S4 and S5, Supporting Information). Scanning electron microscopy (SEM) images revealed that phen‐TPA exhibits a sheet‐like morphology, while phen‐TTA displays a rod‐like morphology (Figure S6, Supporting Information). Nitrogen adsorption‐desorption isotherms were measured to evaluate the porosities of the two COFs. As shown in Figure S7 (Supporting Information), similar isothermal curves were observed for these two COFs. The Brunauer–Emmett–Teller (BET) surface areas of phen‐TPA and phen‐TTA were determined to be 204 and 284 m^2^ g^−1^, respectively. Additionally, pore size distribution analysis using the density functional theory (DFT) model revealed that the pores of phen‐TPA are predominantly centered ≈3.7 nm, while those of phen‐TTA are primarily concentrated at 2.4 nm. These pore sizes are well‐suited for the separation of iodine species. Water contact angle measurements further confirmed the hydrophilic nature of phen‐TPA and phen‐TTA, suggesting their suitability for applications in aqueous environments (Figure S8, Supporting Information). To evaluate the long‐term stability of the COFs, phen‐TPA and phen‐TTA were subjected to 100 h treatments under conditions of strong acid, strong alkali, and high temperature. FT‐IR results indicated that their chemical structures were not significantly affected, and SEM analysis further confirmed that their morphologies remained intact, further validating their exceptional chemical and thermal stability (Figures S9–S12, Supporting Information).

### 2.2 Iodine Adsorption from Aqueous Environment

**Figure 2:**
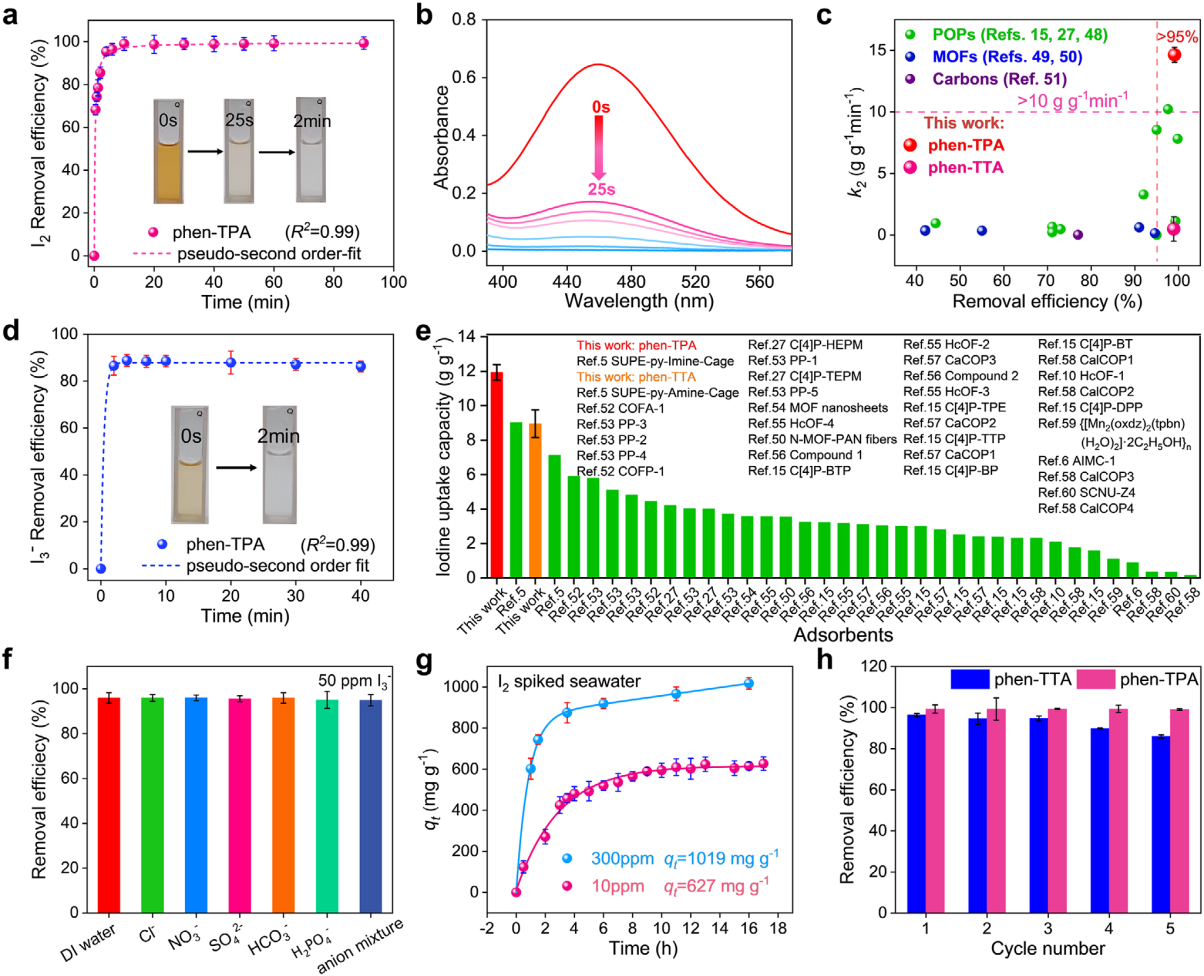
Iodine adsorption from aqueous solutions. a) Time‐dependent adsorption models for phen‐TPA from I_2_ aqueous solution (1.2 mM). b) Time‐dependent UV–Vis spectra upon the addition of phen‐TPA. c) Comparison of I_2_ adsorption kinetic constants of aqueous solution for reported adsorbents.^[^ ^15^, ^27^, ^48^, ^49^, ^50^, ^51^ ^]^ d) Time‐dependent adsorption models for phen‐TPA from I_3_^−^ aqueous solution (0.4 mM). e) Comparison of I_3_^−^ uptake capacities in aqueous solution with various reported adsorbents.^[^ ^5^, ^6^, ^10^, ^15^, ^27^, ^50^, ^52^, ^53^, ^54^, ^55^, ^56^, ^57^, ^58^, ^59^, ^60^ ^]^ f) Static selective iodine adsorption from polluted water with 50 ppm I_3_^−^. g) Time‐dependent adsorption models for phen‐TPA from I_2_‐spiked natural seawater. h) Iodine removal efficiency with recycled phen‐TPA and phen‐TTA obtained from I_2_ aqueous solutions for five cycles.

In the event of a nuclear accident, the release of radioactive iodine into water poses a significant environment threat. Rapid and efficient removal of iodine from water is therefore critical in such emergencies. This study evaluated the adsorption performance of two COFs for iodine removal. Notably, phen‐TPA exhibited a visually perceptible colour change within 25 s of immersion in a 1.2 mM I_2_ aqueous solution. The intensity for the characteristic peak of I_2_ in the (UV–Vis) spectra decreased progressively with adsorption time, reaching equilibrium within 5 min (**Figure** **2**a,b). In contrast, phen‐TTA achieved adsorption equilibrium after 50 min, which, although longer than phen‐TPA, was comparable to other reported iodine adsorbents in aqueous solutions (Figure S13, Supporting Information). Kinetic modelling revealed that the adsorption process for both COFs aligned more closely with a pseudo‐second‐order model, with a correlation coefficient *R^2^* value of 0.9999 and 0.9877 for phen‐TPA and phen‐TTA, respectively, indicating that the rate‐controlling step could involve a chemical interaction process (Figure S14, Supporting Information). The pseudo‐second‐order kinetic constant (*k_2_*) for phen‐TPA was calculated as 14.64 g g^−1^ min^−1^, with an aqueous I_2_ removal efficiency exceeding 99%. Notably, this value reflected the highest adsorption kinetic constant reported for porous adsorbents in I_2_ aqueous solutions (Figure 2c; Table S1, Supporting Information). Furthermore, comparing with the other porous adsorbents, whose iodine adsorption capacity were determined in 1.2 mM aqueous iodine with dosage of 1 mg mL^−1^, the adsorption capacities of phen‐TPA and phen‐TTA were among the better‐performing adsorbents (Figure S15 and Table S2, Supporting Information).

In aqueous solutions, iodine commonly forms polyiodides, with I_3_^−^ being a predominant species.^[^ ^5^, ^15^ ^]^ Both phen‐TPA and phen‐TTA exhibited rapid adsorption, accompanied by clear colour changes within 2 min in 0.4 mM I_3_^−^ aqueous solution (Figure 2d; Figure S16, Supporting Information). Using a concentrated KI/I_2_ aqueous solution (600 mg KI and 300 mg I_2_ in 100 mL H_2_O) containing Cl^−^, Br^−^, NO_3_^−^, and SO_4_^2−^, the maximum iodide adsorption capacity of phen‐TPA and phen‐TTA was ≈11.9 g g^−1^ and 8.9 g g^−1^, respectively. Remarkably, the iodine uptake capacity of phen‐TPA surpassed that of all previously reported materials (Figure 2e; Table S3, Supporting Information). To assess selectivity, competitive adsorption experiments were conducted on phen‐TPA using simulated wastewater from the nuclear industry containing various anions (Cl^−^, NO_3_^−^, SO_4_^2−^, HCO_3_^−^, and H_2_PO_4_^−^). The COF phen‐TPA demonstrated robust resistance to ionic interference, retaining a 95% iodine removal efficiency under these conditions (Figure 2f).

Given the challenges (i.e., extreme pHs and low concentrations) of aqueous iodine adsorption, we further explore iodine removal performance at different pH levels and low concentrations. Iodine removal experiments were carried out using phen‐TPA in iodine‐polluted water at various pH levels. The results showed that phen‐TPA consistently achieved a removal efficiency exceeding 90% in iodine‐polluted water (1.2 mM I_2_ and 0.4 mM I_3_^−^) across a pH range of 2 to 9 (Figure S17, Supporting Information). These findings highlight the excellent performance of phen‐TPA across a wide pH range. Considering that iodine concentrations in real‐world scenarios are typically lower, we next performed adsorption experiments using iodine‐polluted water at a low concentration of 5 ppm.^[^ ^5^, ^44^, ^45^ ^]^ The results confirmed that phen‐TPA effectively removed trace iodine pollutants, achieving a removal efficiency exceeding 91%, highlighting its broad applicability (Figure S18, Supporting Information).

In the event of nuclear accidents, the leakage of radioactive iodine into the surrounding ocean poses a significant challenge due to the complex composition of seawater. As phen‐TPA was expected to exhibit rapid and efficient iodine‐removal capabilities in static aqueous experiments, testing in iodine‐spiked seawater to evaluate its real‐world applicability (Figure 2g). The phen‐TPA achieved a maximum iodine adsorption capacity of 1019 mg g^−1^ within 16 h in natural seawater spiked with 300 ppm I_2_. Furthermore, phen‐TPA reached an equilibrium adsorption capacity of 627 mg g^−1^ in 11 h in seawater containing just 10 ppm I_2_. Given that iodine in natural seawater predominantly exists as iodide, adsorption tests were conducted in different concentrations iodide (KI + I_2_) spiked seawater.^[^ ^46^, ^47^ ^]^ The COF phen‐TPA exhibited an equilibrium adsorption capacity of 2064 mg g^−1^ within 8 h in 300 ppm iodide (KI/I_2_) spiked natural seawater. Remarkably, in 10 ppm of iodide‐spiked natural seawater, phen‐TPA achieved adsorption equilibrium in just 2 h, with an adsorption capacity of 597.9 mg g^−1^ (Figure S19, Supporting Information). Furthermore, iodine removal ability was further explored on phen‐TPA various iodine‐polluted water sources (as low as 5 ppm), including seawater, lake water, tap water, and simulated groundwater (SW). The results showed that phen‐TPA demonstrated excellent iodine removal efficiency, achieving over 90% removal of iodine pollutions in various water sources (Figure S20, Supporting Information). These results highlight phen‐TPA's outstanding potential as an iodine capture material in various water sources, positioning it as a promising solution for radioactive iodine removal.

The reusability of adsorbents is crucial for sustainable and cost‐effective practical applications. For I_2_ capture from aqueous solution, phen‐TPA or phen‐TTA were regenerated by soaking in ethanol with sonication for 2 h, followed by filtration and reuse. Both two COFs demonstrated efficient iodine desorption, with a desorption efficiency exceeding 95% (Figure S21, Supporting Information). The COF phen‐TPA maintained negligible loss in removal efficiency over five reuse cycles, while phen‐TTA retained 86% efficiency after five cycles (Figure 2h). To further evaluate the performance of the phen‐TPA in practical applications, a series of recyclability tests were conducted by adding 100 equivalents of competing anions (Cl^−^, Br^−^, NO_3_^−^, SO_4_^2−^) to iodine‐polluted water. The results demonstrated that after five cycles, phen‐TPA retained an iodine removal efficiency of over 95% for 1.2 mM I_2_ and 92% for 0.4 mM I_3_^−^, even in the presence of 100 equivalent competing anions (Figure S22, Supporting Information). The FT‐IR and SEM analyses of the recycled COFs after five cycles confirmed that their structure and morphology remained intact, highlighting its stability for potential applications (Figures S23 and S24, Supporting Information). These findings underscore the excellent recyclability and reusability of phen‐TPA and phen‐TTA, making them strong candidates for practical applications in iodine removal.

### 2.3 Characterization of Iodine‐Loaded Adsorbents

**Figure 3:**
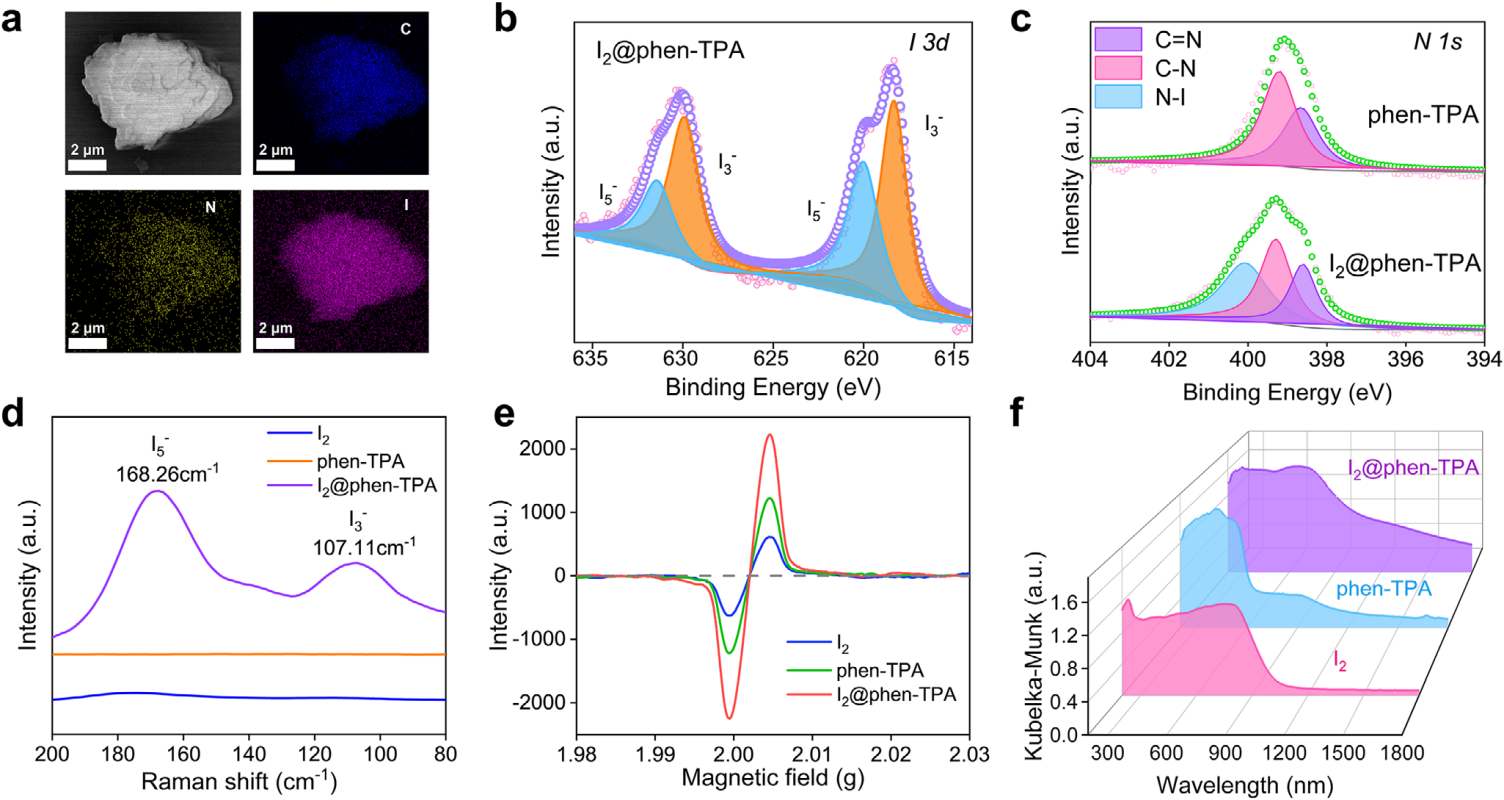
Characterization of iodine‐loaded phen‐TPA. a) SEM images and EDS mapping of iodine‐loaded phen‐TPA. b) High‐resolution XPS for I *3d* of I_2_@phen‐TPA. c) High‐resolution XPS for N *1s* of phen‐TPA and I_2_@phen‐TPA. d) Raman spectra of pristine I_2_, phen‐TPA, and I_2_@phen‐TPA. e) EPR spectra of pristine I_2_, phen‐TPA, and I_2_@phen‐TPA. f) UV–Vis/NIR spectra of I_2_, phen‐TPA, and I_2_@phen‐TPA.

Elemental mapping confirmed the uniform distribution of iodine within the adsorbed phen‐TPA and phen‐TTA (**Figure 3**a; Figure S25, Supporting Information). X‐ray photoelectron spectroscopy (XPS) analysis of phen‐TPA after iodine adsorption (I_2_@phen‐TPA) revealed a distinct I *3d* peak, which was further resolved into four peaks. Peaks at 629.9 and 618.3 eV were attributable to I_3_^−^, while peaks at 631.5 and 620.0 eV were assigned to I_5_^−^ (Figure 3b; Figure S26, Supporting Information). High‐resolution N *1s* XPS spectra of phen‐TPA before and after adsorption showed changes indicative of chemisorption through charge transfer interactions and complexes formation. Before adsorption, the spectrum displayed two peaks, which expanded to three after adsorption, confirming interactions between iodine and nitrogen sites. The electron‐rich N sites facilitated charge transfer, converting I_2_ into I_3_^−^ and I_5_^−^. The main iodine species on the absorbents was identified as I_3_^−^ based on the peaks area analysis. Furthermore, a new N–I bond peak appeared at 400.6 eV in the N *1s* spectrum, and shifts in C═N and C─N positions from 398.7 to 398.9 eV and 399.2 to 399.6 eV, respectively, further validated the formation of charge transfer complexes (Figure 3c). Similarly, XPS analysis of I_2_@phen‐TTA showed I *3d* peaks corresponding to I_3_^−^ and I_5_^−^, along with a new N─I peak in the N *1s* spectrum, confirming charge transfer interactions between iodine and N sites of phen‐TTA (Figure S27, Supporting Information). The COF phen‐TPA also displayed new I *3d* peaks after iodine capture from seawater, confirming the presence of I_3_^−^ and I_5_^−^ (Figure S28, Supporting Information). These results indicate that iodine adsorption in phen‐TPA and phen‐TTA, particularly in iodine‐spiked seawater, is primarily driven by the formation of charge transfer complexes.

Raman spectroscopy confirmed the presence of adsorbed iodine species. A comparison of the spectra for pure I_2_, phen‐TPA, and I_2_@phen‐TPA revealed two distinct bands at 107.1 and 168.3 cm^−1^ in I_2_@phen‐TPA, corresponding to I_3_^−^ and I_5_^−^, respectively (Figure 3d). Similarly, in I_2_@phen‐TTA, two bands at 108.5 and 165.7 cm^−1^ were attributed to I_3_^−^ and I_5_^−^, respectively (Figure S29, Supporting Information). Electron paramagnetic resonance (EPR) spectroscopy provided further evidence for charge transfer complexes in iodine adsorption. The multiple electron‐rich N‐sites in phen‐TPA and phen‐TTA facilitated charge transfer to iodine, generating a much stronger paramagnetic signal in I_2_@phen‐TPA and I_2_@phen‐TTA compared to pristine I_2_, phen‐TPA, and phen‐TTA (Figure 3e; Figure S30, Supporting Information). UV–Vis/NIR absorption spectra of pristine I_2_ and phen‐TPA displayed weak peaks beyond 1000 nm, while I_2_@phen‐TPA exhibited a broad absorption peak from 200 to 1400 nm. The adsorption in the NIR region was primarily attributed to the formation of charge transfer complexes (Figure 3f). In contrast, I_2_@phen‐TTA exhibited weaker NIR adsorption, likely due to the limited formation of charge transfer complexes between phen‐TTA and iodine (Figure S31, Supporting Information). These spectral analyses confirmed that phen‐TPA effectively captured and retained iodine species via charge transfer interactions at multiple N‐sites, while phen‐TTA demonstrated comparatively lower iodine adsorption efficiency.

### 2.4 Iodine Adsorption Mechanism

**Figure 4:**
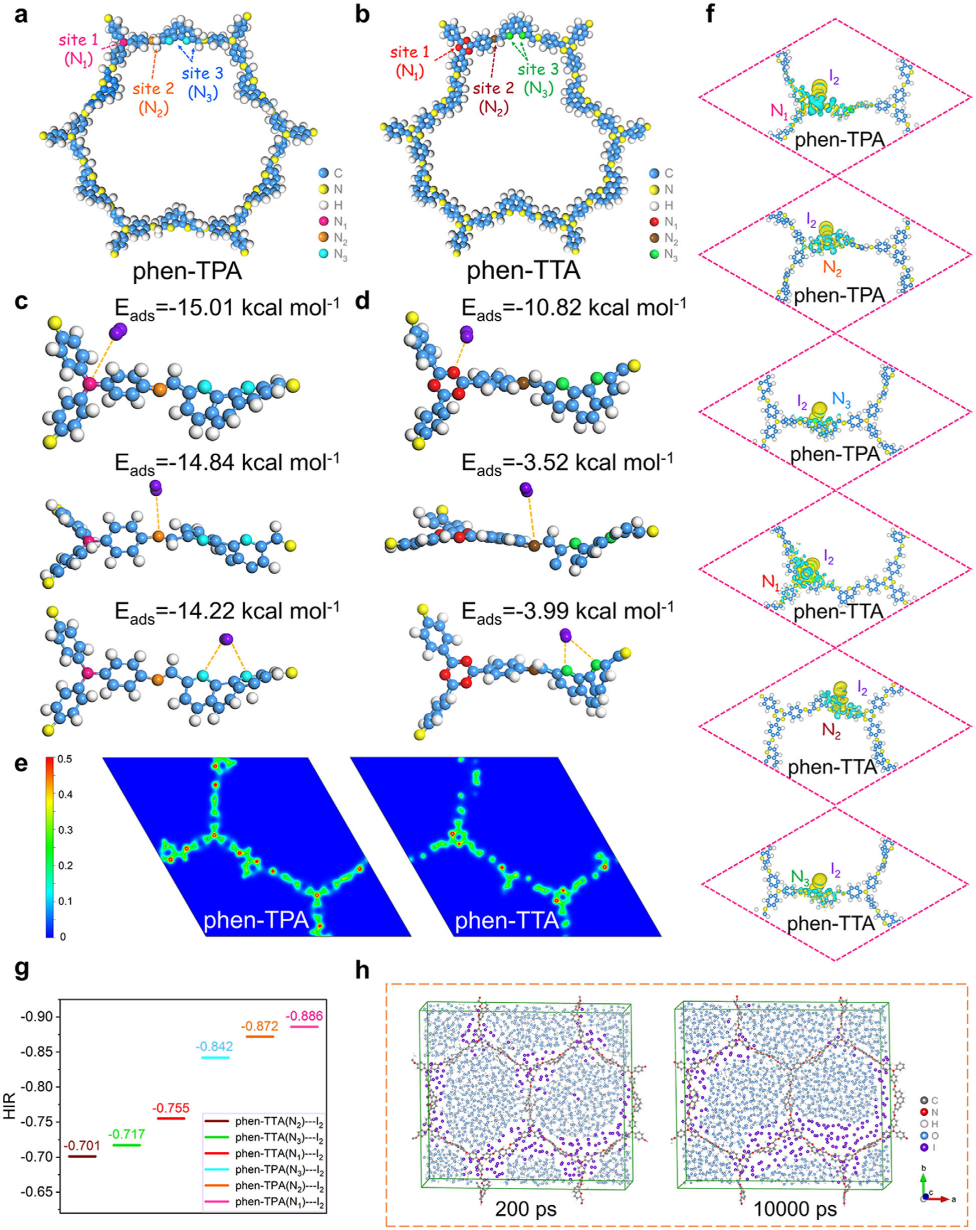
Interaction mechanism of phen‐TPA and phen‐TTA with iodine. a) Diagram of the N‐sites of phen‐TPA. b) Diagram of the N‐sites of phen‐TTA. c) Three configurations of I_2_ interacting with the N‐sites in phen‐TPA and the corresponding calculated adsorption energies. d) Three configurations of I_2_ interacting with the N‐sites in phen‐TTA and the corresponding calculated adsorption energies. e) Local charge density maps of phen‐TPA and phen‐TTA. f) Calculated electron density isosurfaces of the I_2_ and *N_1_*, *N_2_*, *N_3_* of phen‐TPA and phen‐TTA, respectively. g) Hirshfeld charge population of phen‐TPA and phen‐TTA (negative values denote the loss of electrons). h) Molecular dynamics simulation of phen‐TPA at 200 and 10 000 ps, respectively.

To better understand the iodine adsorption mechanisms of charge properties of N‐rich COFs, and to uncover the underlying factors influencing adsorption capacity and rate differences between phen‐TPA and phen‐TTA, a series of theoretical simulations were evaluated (**Figure** **4**). As shown in Figure 4a,b, amine and phenanthroline were defined as *N_1_* and *N_3_* in phen‐TPA, while triazine and phenanthroline were designated as *N_1_* and *N_3_* in phen‐TTA, respectively. Additionally, the nitrogen sites in C═N bonds formed via Schiff base reactions identified as *N_2_*, contributing to charge transfer complex formation with iodine species. DFT simulations were employed to calculate the binding energies between iodine species (I_2_ and I_3_^−^) and the N‐sites involved in adsorption. For phen‐TPA, *N_1_* exhibited had the strongest adsorption potential for I_2_, with a binding energy of −15.01 kcal mol^−1^, followed by *N_2_* (−14.84 kcal mol^−1^) and *N_3_* (−14.22 kcal mol^−1^) (Figure 4c). In contrast, *N_1_*, *N_2_* and *N_3_* of phen‐TTA displayed significantly weaker binding energies of −10.82, −3.52, and −3.99 kcal mol^−1^, respectively (Figure 4d). The superior binding energies in phen‐TPA were consistent with experimental findings, demonstrating its rapid and efficient iodine capture. Interestingly, despite *N_2_* and *N_3_* in both COFs originating from the same linker or monomer, phen‐TPA displayed binding energies approximately four times stronger than phen‐TTA. This difference was attributed to the enhanced local charge density at *N_1_* sites in phen‐TPA, which influenced the overall charge distribution within the framework. In order to confirm this speculation and further explored the core mechanism of the relationship between N sites and iodine adsorption capacities, the charge features of N sites were calculated and investigated. Charge density analyses indicated higher local charge densities (orange regions) for *N_1_, N_2_ and N_3_* in phen‐TPA compared to phen‐TTA (Figure 4e). Bader charge populations further confirmed this, showing an effective charge occupancy of 0.98 for *N_1_* in phen‐TPA, significantly higher than the 0.06 observed for *N_1_* in phen‐TTA. Notably, *N_2_* and *N_3_* sites exhibited similar charge occupancies in both COFs (Table S4, Supporting Information). These findings confirmed that the superior binding energies for iodine adsorption at *N_2_* and *N_3_* in phen‐TPA, compared to phen‐TTA, were primarily driven by the enhanced local charge density at *N_1_* sites. Moreover, DFT simulations indicated that the binding energy trends for I_3_⁻ mirrored those for I_2_ (Figure S32, Supporting Information). Notably, *N_1_* and *N_2_* in phen‐TPA exhibited particularly strong binding energies with I_3_⁻, explaining why phen‐TPA is the most effective adsorbent for I_3_⁻ capture reported to date.

Considering the spectral characterization results indicating the formation of charge transfer complexes during the chemisorption of I_2_ by phen‐TPA and phen‐TTA, the adsorption capacity of these COFs for I_2_ was further analyzed using differential charge density and Hirshfeld charge population. As shown in Figure 4f, the differential charge densities of I_2_ absorbed on phen‐TPA and phen‐TTA were calculated. The 3D charge density isosurfaces of the N‐sites and I_2_ revealed that during chemisorption, the N‐sites in both COFs lost electrons, while I_2_ gained electrons. The Hirshfeld charge population quantitatively assessed the extent of charge transfer during chemisorption (Figure 4g). For phen‐TPA, *N_1_, N_2,_* and *N_3_* exhibited enhanced charge transfer interactions, with total transferred charges of 0.886, 0.872, and 0.842, respectively, from N atoms to iodine. In constrast, *N_1_, N_2_* and *N_3_* in phen‐TTA showed lower charge transfer value of 0.755, 0.701, and 0.717, respectively. This confirmed the charge transfer mechanism during I_2_ adsorption, where electrons are transferred from the N‐sites of phen‐TPA and phen‐TTA to iodine. Among these sites, *N_1_* of phen‐TPA, which donated the most charge, exhibited the strongest iodine adsorption energy.

The kinetics of aqueous iodine removal depended not only on binding energy but also on the diffusion coefficient of I_2_ in water. Molecular dynamics models of I_2_ diffusion in the presence of water within the phen‐TPA and phen‐TTA frameworks were simulated (Figure 4h; Figure S33, Supporting Information). The results demonstrated that water molecules were randomly distributed within the framework, and iodine molecules preferentially adsorbed near the N‐sites. Overtime, I_2_ freely diffused within the frameworks until adsorption equilibrium was reached. When the mean square displacement (MSD) of I_2_ molecules in phen‐TPA and phen‐TTA was calculated over a molecular motion of 20,000 ps, the diffusion constant of I_2_ in phen‐TPA consistently surpassed that in phen‐TTA by ≈1.5‐fold (Figure S34, Supporting Information). This indicated that phen‐TPA reached equilibrium more rapidly than phen‐TTA, highlighting its superior kinetic adsorption performance, in agreement with experimental observations.

Theoretical calculations offer a clear explanation for the exceptional performance of phen‐TPA in rapid iodine removal kinetics and breakthrough iodine adsorption capacity. The N‐rich phen‐TPA adsorbs iodine through charge transfer, while the enhancement of local charge density optimizes the overall charge distribution of the framework. This results in more effective charge transfer at multiple nitrogen sites during iodine adsorption, leading to significantly stronger iodine binding energy and increased adsorption capacity. Molecular dynamics simulations further reveal that phen‐TPA reaches adsorption equilibrium faster in aqueous environments, with a diffusion coefficient 1.5 times greater than that of phen‐TTA, confirming its superior removal kinetics. These results provide strong theoretical support for phen‐TPA's high adsorption capacity and excellent removal kinetics, demonstrating the effectiveness of the local charge density enhancement strategy in improving adsorption performance.

### 2.5 Large‐Scale Dynamic Column Breakthrough Adsorption

**Figure 5:**
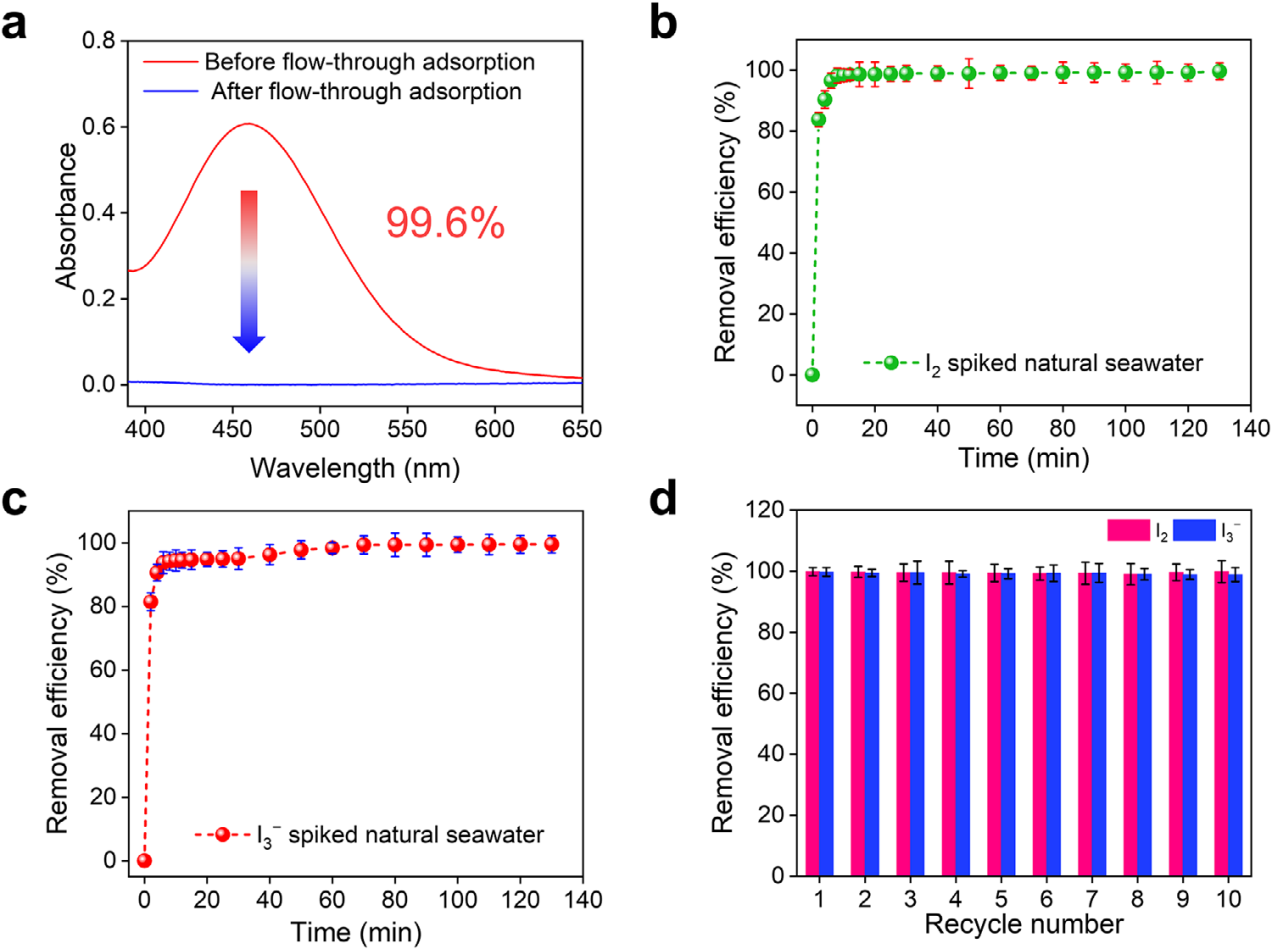
Dynamic column breakthrough test for iodine separation. a) UV–Vis spectra of I_2_ aqueous solutions before and after flow‐through adsorption by phen‐TPA in 1.2 mM I_2_ spiked simulated nuclear wastewater. b) Time‐dependent dynamic aqueous I_2_ separation from 1.2 mM I_2_ spiked natural seawater. c) Time‐dependent dynamic aqueous I_3_^−^ separation from 1.2 mM I_3_^−^ spiked natural seawater. d) I_2_ and I_3_^−^ removal efficiency over 10 cycles column breakthrough adsorption in I_2_ or I_3_^−^ spiked natural seawater.

For potential industrial applications, the iodine removal capability of scale‐up fabricated phen‐TPA was evaluated in simulated nuclear wastewater and iodine‐polluted seawater using a dynamic column breakthrough test with automatic integrated flow adsorption equipment. The COF phen‐TPA was packed into an adsorption column, and the simulated nuclear wastewater as well as iodine‐spiked seawater was pass through to assess the separation performance. For each column, simulated nuclear wastewater with an initial I_2_ concentration of 1.2 mM was flowed through at a flow rate of 3 L h^−1^. The yellow I_2_ aqueous solution turned completely colourless after passing through the column, achieving a removal efficiency of 99.6% (**Figure** **5**a). In 1.2 mM iodine‐polluted seawater, 8 L of natural seawater spiked with either I_2_ or I_3_^−^ was passed through a column containing 20 g of phen‐TPA at a flow rate of 3 L h^−1^. The results demonstrated high removal efficiencies of ≈99.5% for both iodine species in seawater (Figure 5b,c). The reusability of the phen‐TPA was determined in I_2_ and I_3_^−^ spiked natural seawater over 10 reuse cycles. Even after 10 cycles, phen‐TPA retained high iodine removal efficiencies of 99% and 98%, respectively, indicating excellent practical reusability (Figure 5d). These findings demonstrate the high dynamic iodine separation performance of phen‐TPA, highlighting its strong potential for treating aqueous iodine pollution in the nuclear industry.

## 3 Conclusion

In summary, we report two N‐rich COFs, phen‐TPA and phen‐TTA, featuring highly exposed multiple iodine binding sites with varying charge densities for the removal of iodine from aqueous solutions. Taking advantage of multiple iodine binding sites and enhanced local charge density at N‐sites, the optimized COF phen‐TPA exhibits exceptional aqueous iodine capture performance, including a record‐breaking rapid removal kinetic constant for I_2_ in aqueous solution (14.64 g g^−1^ min^−1^) and the highest reported adsorption capacity for aqueous I_3_^−^ (11.9 g g^−1^). In contrast, despite having high‐exposure multiple N‐sites, phen‐TTA shows moderate iodine capture capacity in aqueous solutions. Theoretical calculations reveal that enhanced charge density at the N‐sites is the critical factor in improving iodine binding energy. This enhancement facilitates greater charge transfer during chemisorption, enabling phen‐TPA to achieve efficient and rapid aqueous iodine removal. This study elucidates a fundamental mechanism where localized charge density enhancement at nitrogen sites significantly boosts iodine adsorption performance, enabling remarkable capture and rapid removal of aqueous iodine. Furthermore, we have added the large‐scale dynamic column breakthrough adsorption via automatic integrated flow adsorption equipment. The COF phen‐TPA realizes a high dynamic removal ability of 99.5% to aqueous iodine. These findings offer a promising strategy for designing high‐performance iodine adsorbents and provide an innovative approach for addressing iodine pollution, particularly in nuclear emergency scenarios.

## Conflict of Interest

The authors declare no conflict of interest.

## Supporting information



Supporting Information

## Data Availability

The data that support the findings of this study are available from the corresponding author upon reasonable request.
