# Supplementary material for: Local Charge Density Enhancement Strategy in Nitrogen‐rich Covalent Organic Framework for Boosted Iodine Removal From Water
Source: Adv Sci (Weinh). 2025 May 20;12(30):e00697. doi: 10.1002/advs.202500697 (PMC12376611; doi:10.1002/advs.202500697)
Supplement: Supplementary file 1 — Supporting Information [file ADVS-12-e00697-s001.docx]

Supporting Information

Local Charge Density Enhancement Strategy in Nitrogen-rich Covalent Organic Framework for Boosted Iodine Removal from Water

*Yue Ma^1,2^, Jinjiao Pan^1,2^, Huazhen Rong^1^, Lu Liu^1^, Yilei Zhang^1^, Xuewen Cao^1^, Jiacheng Zhang^1^, Tao Liu^1^, Yihui Yuan^1^*, and Ning Wang^1^**

Dr. Y. Ma, J. Pan, Dr. H. Rong, L. Liu, Y. Zhang, X. Cao, J. Zhang, Prof. T. Liu, Prof. Y. Yuan, Prof. N. Wang

^1^State Key Laboratory of Marine Resource Utilization in South China Sea, Hainan University, Haikou 570228, P. R. China

^2^These authors contributed equally: Yue Ma, Jinjiao Pan

*Corresponding authors

Email: wangn02@foxmail.com; yuanyh@hainanu.edu.cn

**Materials**

All reactants and solvents used in this study were purchased from commercial suppliers with no further purification. The aqueous solution of iodine was prepared using deionised water, seawater was collected from the west coast of Haikou, Hainan, and filtered through a 0.22 μm filter membrane.

**Characterization**

^13^C cross-polarization magicangle spinning (CP/MAS) NMR spectroscopy were recorded using a Bruker BioSpin GmbH instrument. UV-Vis spectra were recorded using a UV-1600 spectrophotometer. The morphology was determined using a Zeiss Sigma500 field launch scanning electron microscope. Fourier transform infrared (FT-IR) spectra were recorded using a PerkinElmer Fourier transform infrared spectrometer. A Verios G4 UC field-emission scanning electron microscope (SEM) was used to observe the surface morphologies of the two prepared adsorbents. N_2_ adsorption and desorption at 77 K were determined using an American Mike ASAP2460 automatic. The two adsorbents were degassed at 120°C under vacuum for 12 h before the N_2_ adsorption and desorption analyses. X-ray photoelectron spectra (XPS) was performed using a Thermo SCIENTIFIC Nexsa spectrometer. The UV/Vis/NIR spectra were recorded using a UH4150 spectrophotometer (Direct Light Detector). Electron paramagnetic resonance (EPR) spectroscopy was performed on a Bruker A300-10/12 spectrometer. X-ray diffraction (XRD) measurements were performed using a Rigaku Smart Lab X-ray diffractometer. Raman tests were performed using a Renishaw PLC laser Raman spectrometer. Inductively coupled plasma (ICP-MS) measurements were performed using a Thermo Scientific ICAP RQ ultrasensitive inductively coupled plasma emission mass spectrometer.

Experimental Section

*Synthesis of COFs*: To fabricate COF phen-TPA and phen-TTA, 1,10-phenanthrolin-2,9-diformaldehyde (phen) (0.06 mmol) and tris(4-aminophenyl) amine (TPA) (0.04 mmol) or tris(4-aminophenyl) triazine (TTA) (0.04 mmol) were dissolved in 2 mL of tetrahydrofuran (THF) (or 1.6 mL of mesitylene and 0.4 mL 1,4-dioxane for phen-TTA). Finally, 0.2 mL of 6 M aqueous acetic acid was added. The reactor was sealed and heated at 120°C for 5 days, yielding a brick red or brown powder that was isolated by filtration and washed with *N*, *N*-dimethylformamide (DMF), THF, and dichloromethane. The resulting power was dried at 50°C at vacuum overnight. The yields of phen-TPA and phen-TTA are 68% and 57%, respectively.

*Static iodine adsorption from aqueous solution:* The adsorbent (5 mg) was immersed in an aqueous iodine solution (I_2_: 1.2 mM, 5 ppm; I_3_^−^: 0.4 mM, 5 ppm) (5 mL), and stirred at a rate of 1000 rpm at room temperature, sampled at different times, and filtered using a 0.22 μm polyether sulfone (PES) membrane. The residual iodine concentration in the filtrate was measured using UV-Vis spectroscopy (I_2_: 1.2 mM; I_3_^−^: 0.4 mM) and ICP-MS (5 ppm I_2_ and I_3_^−^).

All adsorption experiments were conducted with more than three parallel trials and were carried out in dark conditions to avoid the effects of light exposure. During the experiment, brown glassware should be used and wrapped in tinfoil to block light, particularly ultraviolet radiation, which can decompose iodine or interfere with the adsorption process.

The removal efficiency of iodine was calculated as

$$\frac{C_{0}-C_{t}}{C_{0}}\times100\%$$

Where $C_{0}$ (mM) and $C_{t}$ (mM) are the iodine concentrations before and after adsorption, respectively.

The capture capacity of iodine was calculated as

$q_{t}$=$\frac{{(C}_{0}-C_{t}) M_{w} V}{m}$

Where $q_{t}$ (g g^−1^) represents the amount of iodine adsorbed per gram of adsorbent at time$t$ (min). $C_{0}$(mM) and $C_{t}$ (mM) are the initial and residual concentrations of iodine in the stock solution and filtrate, respectively, $V$ (L) is the volume of the iodine solution, and $m$ (g) is the mass of the adsorbent used in the study. $M_{w}$ (g mol^−1^) is the molar mass of iodine.

The kinetic model of adsorption experiment was fitted using pseudo-first-order kinetic model, expressed as

$$\log\left( q_{e}-q_{t} \right)=\log q_{e}-\frac{k_{1}}{2.303}t$$

Where $q_{e}$ (g g^−1^), $q_{t}$ (g g^−1^), $t$ (min), $k_{1}$ (min^−1^) represent the iodine adsorption capacity at equilibrium, the iodine adsorption capacity at $t$, sampling time and pseudo-first-order kinetic constant, respectively.

The kinetic models of the adsorption experiments were Ho and McKay pseudo-second-order kinetic model, expressed as

$$\frac{t}{q_{t}}=\frac{t}{q_{e}}+\frac{1}{k_{2}q_{e}^{2}}$$

Where $q_{e}$ (g g^−1^) represents the amount of iodine adsorbed at equilibrium, $q_{t}$ (g g^−1^) represents the amount of iodine adsorbed at $t$ (min), $k_{2}$(g g^−1^min^−1^) is pseudo-second-order kinetic constant, which was calculated by the intercept and slope of the corresponding curve with $\frac{t}{q_{t}}$ as the y axis and $t$ as the x axis.

*Static iodine adsorption from aqueous solution at different pH ranges:* The pH was adjusted using hydrochloric acid and sodium hydroxide aqueous solutions in 1.2 mM I_2_ or 0.4 mM I_3_^−^ aqueous solutions. 5 mg phen-TPA was added to 5 mL I_2_ or I_3_^−^ aqueous solution with pH = 2−9, stirred at a rate of 1000 rpm at room temperature, sampled, and filtered using a 0.22 μm PES membrane at different times. The residual iodine concentration in the filtrate was measured by ICP-MS.

*Static iodine adsorption from various water sources:* For adsorption performance assay in wastewater, the adsorbent was immersed in 100 mL KI/I_2_ aqueous solution (600 mg KI +300 mg I_2_ + 100 mL H_2_O) in the presence of 100 equivalents of equal-molar Cl^−^, Br^−^, NO_3_^−^, and SO_4_^2−^ and stirred at a rate of 1000 rpm at room temperature for 48 h. After 48 h, the material was filtered using a 0.22 μm PES membrane and washed with deionized water until the filtrate turned clear and transparent. The filtrate was collected, 2% starch indicator was added and then titrated with a 0.05 M sodium bisulfite solution. The remaining iodine concentration in the filtrate was calculated by titration. The adsorption selectivity assay was performed in 50 ppm I_3_^−^ solution with the presence of equal-molar interfering anions, including Cl^−^, NO_3_^−^, SO_4_^2−^, HCO_3_^−^, and H_2_PO_4_^−^.

For adsorption performance assay in seawater, the adsorbent was added to the filtered natural seawater supplemented with 10 and 300 ppm I_2_ (or I_3_^−^), stirred at a rate of 1000 rpm at room temperature, sampled, and filtered at different times. The residual iodine concentration in the filtrate was measured using ICP-MS.

For removal ability assay in various water sources, the adsorbent was added to the filtered seawater, lake water, tap water and simulated groundwater (SW) supplemented with 5 ppm I_2_ and I_3_^−^ aqueous solutions, stirred at a rate of 1000 rpm at room temperature, sampled, and filtered using a 0.22 μm PES membrane at different times. The residual iodine concentration in the filtrate was measured using ICP-MS. SW is made according to the following distribution: silicic acid (H_2_SiO_3_∙nH_2_O) 15.3 mg L^−1^, potassium chloride (KCl) 8.2 mg L^−1^, magnesium carbonate (MgCO_3_) 13.0 mg L^−1^, sodium chloride (NaCl) 15.0 mg L^−1^, calcium sulfate (CaSO_4_) 67.0 mg L^−1^, calcium carbonate (CaCO_3_) 150.0 mg L^−1^.^[1]^

*Recyclable performance assay:* The 5 mg adsorbents (phen-TPA and phen-TTA) were immersed in the 5 mL I_2_ aqueous solution (1.2 mM), stirred at a rate of 1000 rpm at room temperature, sampled at different times, and filtered using a 0.22 μm PES membrane until the concentration of iodine remaining in the filtrate remained unaltered. The residual iodine concentration in the filtrate was measured using UV-Vis spectroscopy and ICP-MS and filtered to obtain I_2_@phen-TPA and I_2_@phen-TTA. Thereafter, I_2_@phen-TPA and I_2_@phen-TTA were added to a beaker filled with ethanol and ultrasonicated for 2 h. The regenerated samples were filtered and vacuum-dried at 40°C. The recycled adsorbent was used in subsequent cycles at least five times.

For further evaluate the ion interference resistance of phen-TPA during the process of recycling, 5 mg phen-TPA was immersed in the 5 mL I_2_ aqueous solution (1.2 mM) and I_3_^−^ aqueous solution (0.4 mM I_3_^−^) containing 100 equivalents of competing anions (Cl^−^, Br^−^, NO_3_^−^, SO_4_^2−^), with the experimental procedure remaining consistent with the aforementioned cyclic tests.

*Dynamic flow-through experiment:* The adsorbent (20 g) was placed in the filling column. The simulated nuclear wastewater contained aqueous I_2_ (1.2 mM) and I_2_ (or I_3_^−^) spiked natural seawater (1.2 mM) was flowed through the column by a metering pump at a flow rate of 3 L h^−1^. The solution flowing through the sample-filled column was collected, and the remaining iodine concentration in the solution was measured using UV-Vis spectroscopy and ICP-MS.

*Computational method:* The adsorption energies of I_2_ in the COFs were determined using the CP2K package (version 7.1) in the framework of DFT based on the hybrid Gaussian and plan-wave schemes.^[2,3]^ The molecular orbitals of the valence electrons were expanded into DZVP-MOLOPT-SR-GTH basis sets, while the atomic core electrons were described using Goedecker-Teter-Hutter (GTH) pseudopotentials.^[4,5]^ A plane-wave density cut-off of 500 Ry was adopted. Long-range van der Waals interactions were described by the DFT-D3 approach.^[6]^ All structures were fully relaxed by CP2K with the BFGS scheme, and the force convergence criterion was set to 4.5×10^−4^ hartree bohr^-1^.

Molecular dynamic analysis was performed using the GROMACS software package (version 2021.3).^[7-10]^ The system was constructed using packmol, and the SPC water model was filled into the matrix to simulate a solution environment.^[11,12]^ Atomic interactions were parameterised using the general AMBER force field (GAFF).^[13]^ After energy minimisation, a production run was performed in an NVT ensemble at 300 K with a time step of 1 fs. The temperature of the system was controlled by a v-rescale thermostat (τT = 1 ps). After 20 ns of simulation, the MSD of the I_2_ particles were analysed using the GROMACS toolkit.


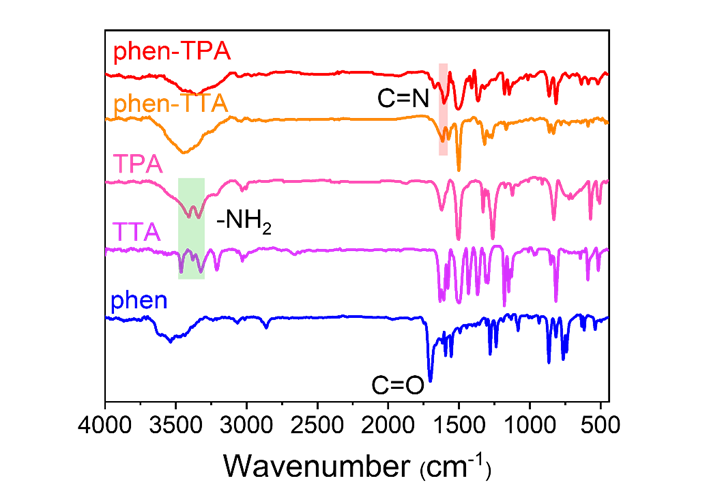


**Figure S1.** FT-IR spectra of phen-TPA and phen-TTA.


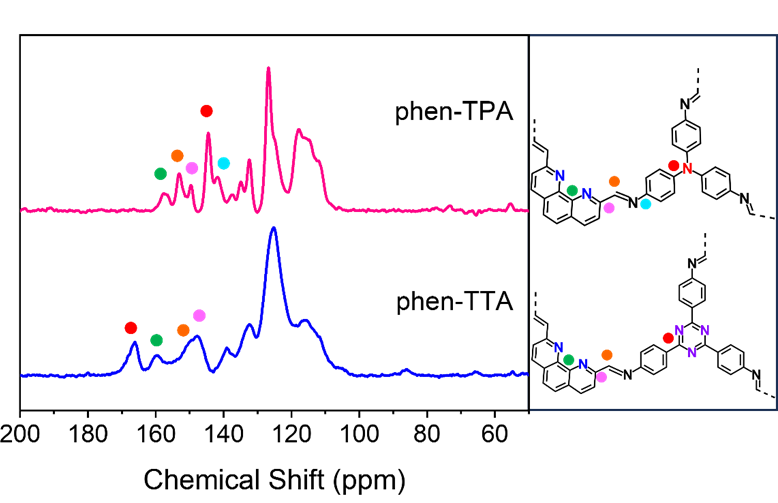


**Figure S2.** ^13^C NMR spectra of phen-TPA and phen-TTA.


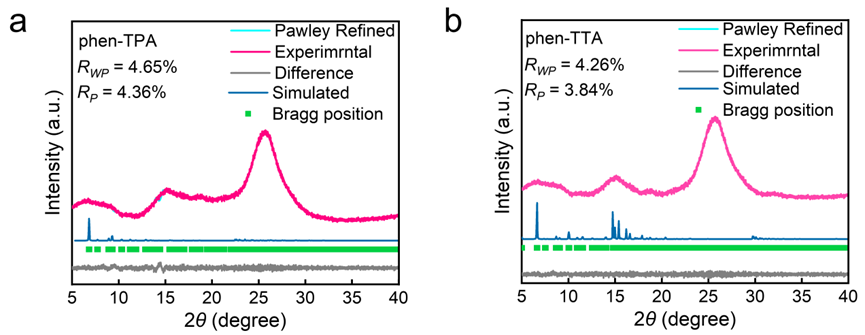


**Figure S3.** PXRD characterizations of COFs. a) phen-TPA and b) phen-TTA.

**
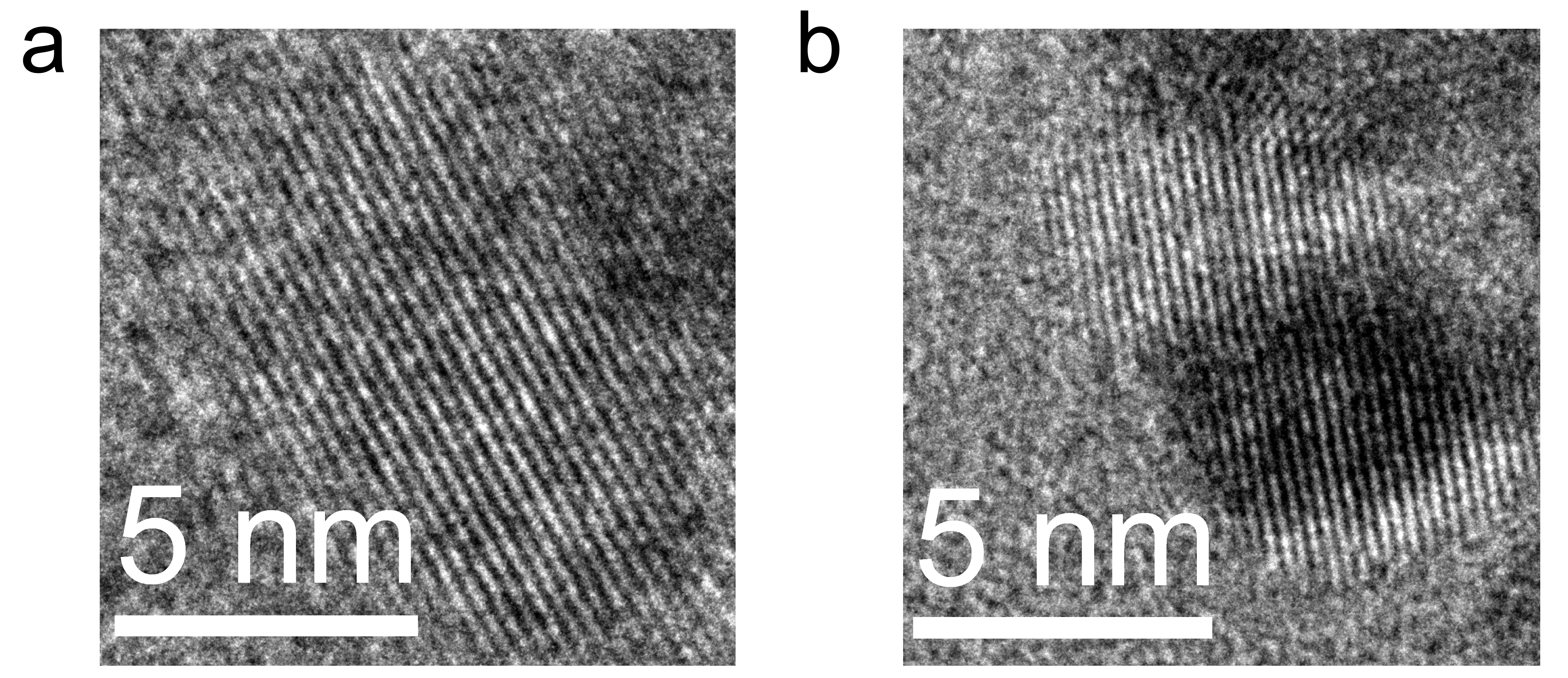
**

**Figure S4.** HRTEM images of COFs. a) phen-TPA and b) phen-TTA.


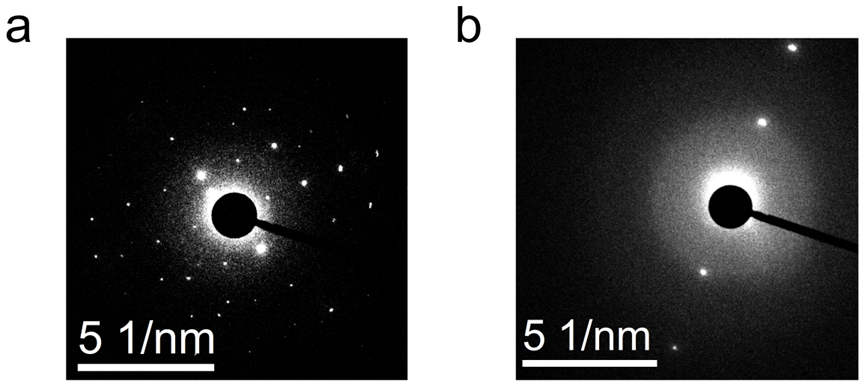


**Figure S5.** SAED image of COFs. a) phen-TPA and b) phen-TTA.


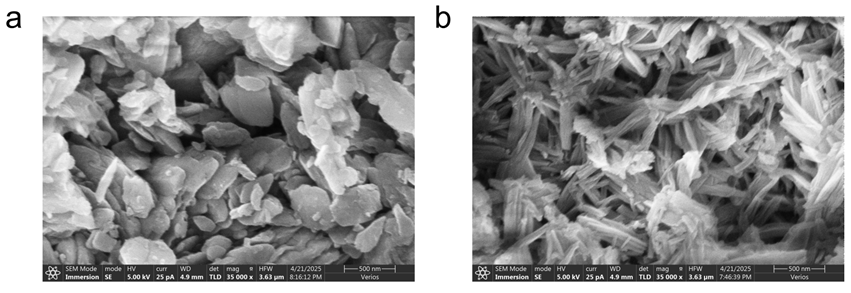


**Figure S6.** SEM images of COFs. a) phen-TPA. b) phen-TTA.

**
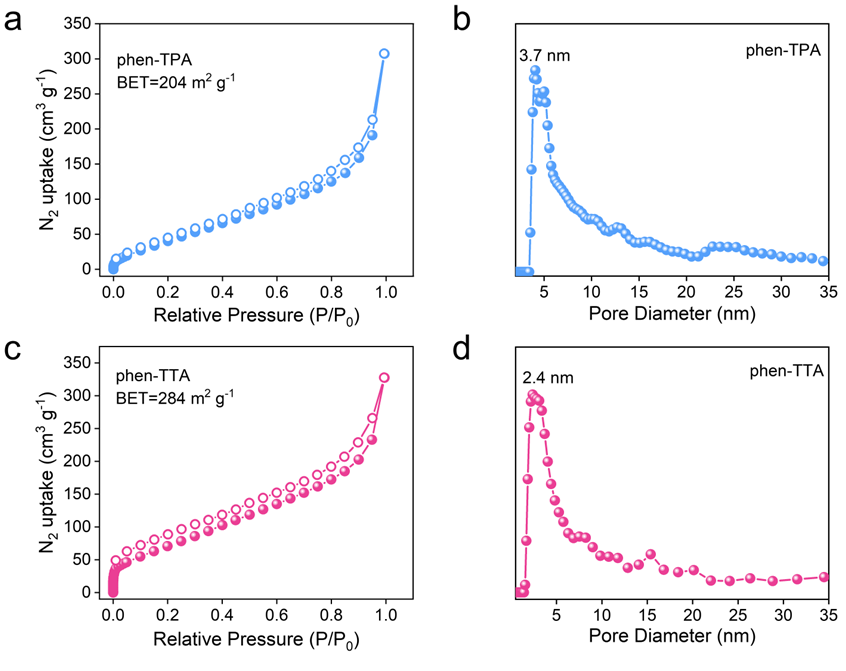
**

**Figure S7.** N_2_ adsorption-desorption isotherm and pore size distribution of COFs at 77k. a) b) phen-TPA and c) d) phen-TTA.


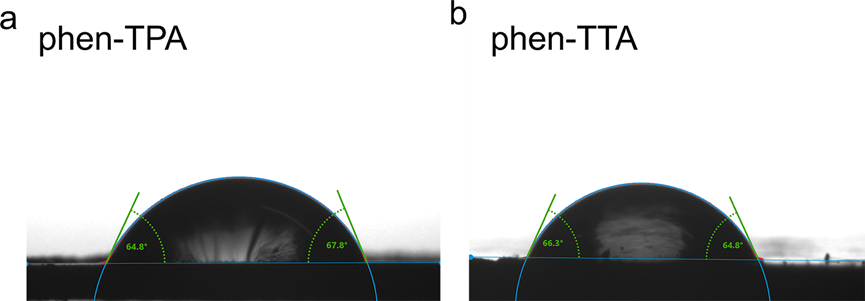


**Figure S8.** Water contact angle of two COFs. a) phen-TPA and b) phen-TTA.


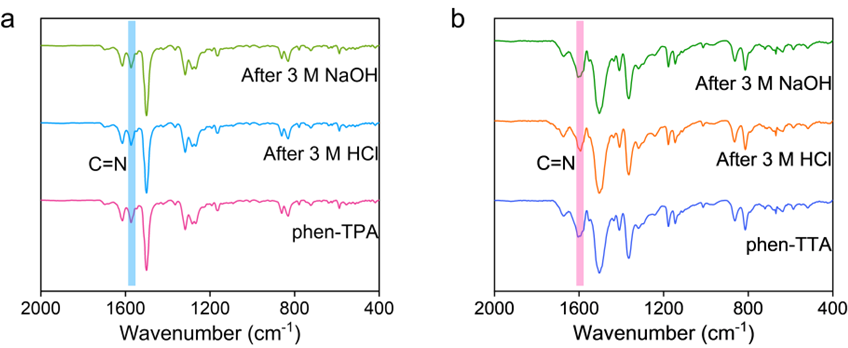


**Figure S9.** FT-IR spectra of COFs after being soaked in 3 M HCl and 3 M NaOH for 100 hours, respectively. a) phen-TPA. b) phen-TTA.


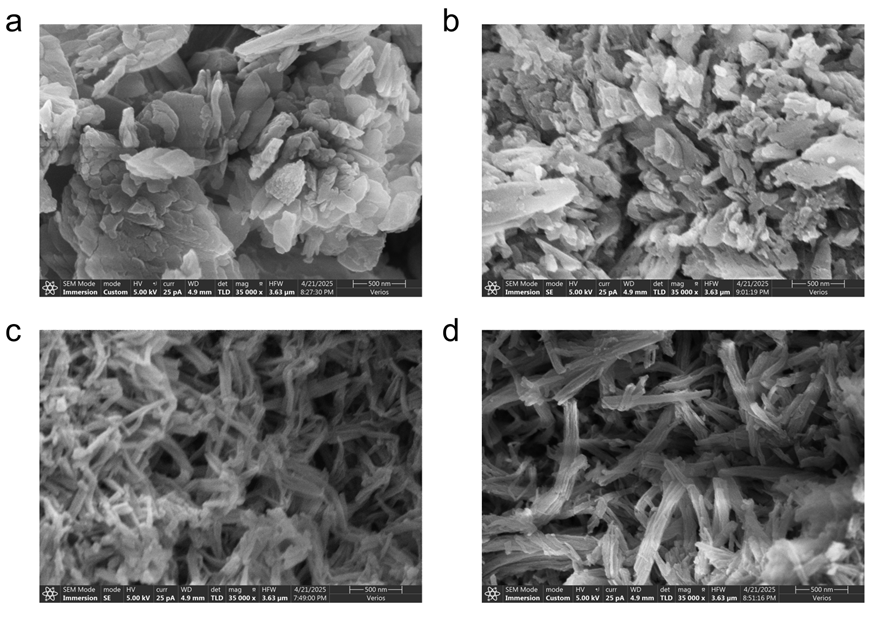


**Figure S10.** SEM images of COFs after being treated with acid and alkali for 100 hours. a) phen-TPA after being soaked in 3 M HCl. b) phen-TPA after being soaked in 3 M NaOH. c) phen-TTA after being soaked in 3 M HCl. d) phen-TTA after being soaked in 3 M NaOH.


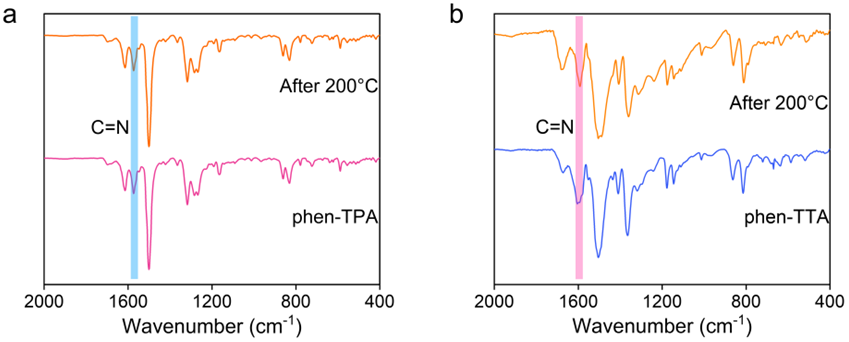


**Figure S11.** FT-IR spectra of COFs after being placed at 200℃ for 100 hours. a) phen-TPA. b) phen-TTA.


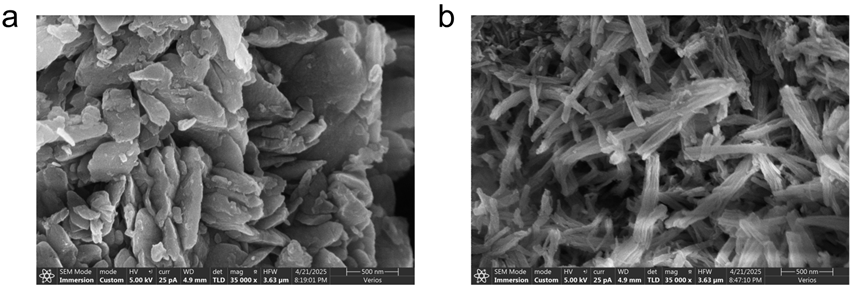


**Figure S12.** SEM images of COFs after being placed at 200℃ for 100 hours. a) phen-TPA. b) phen-TTA.


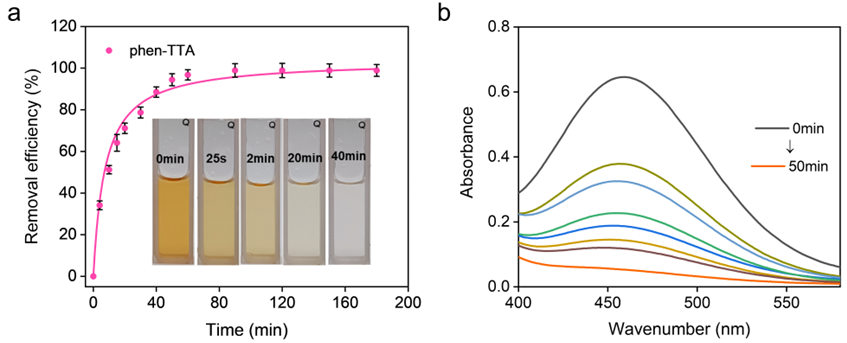


**Figure S13.** Adsorption of iodine by phen-TTA in 1.2 mM I_2_ aqueous solution. a) Time dependent removal efficiency (inset: color changed of iodine solution during adsorption). b) Corresponding UV-Vis spectra of the I_2_ aqueous solution.


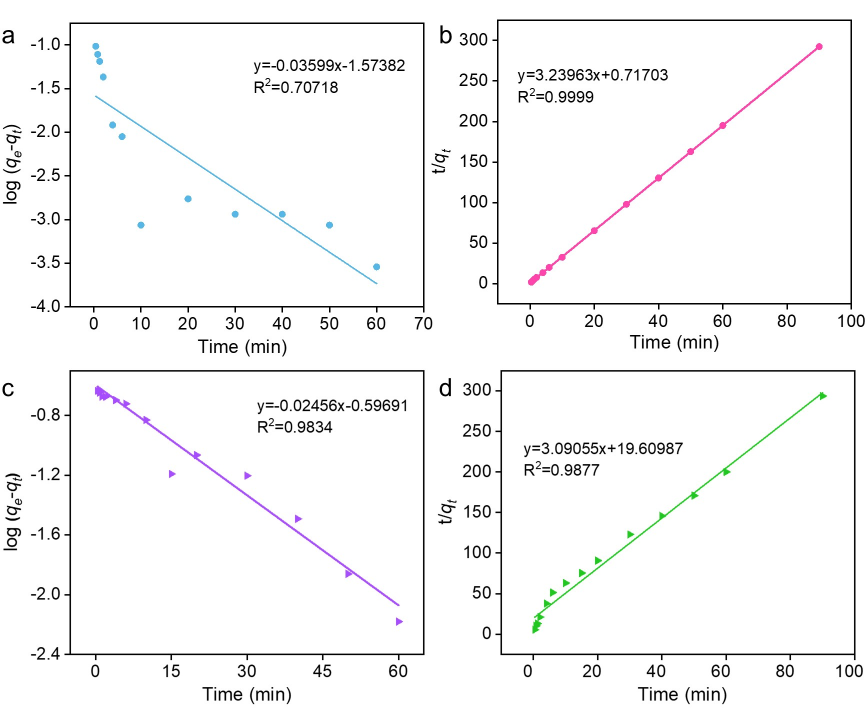


**Figure S14.** Linear fitting of the adsorption kinetic of phen-TPA and phen-TTA to iodine in 1.2 mM I_2_ aqueous solution. a) Pseudo-first-order kinetic linear fitting of phen-TPA. b) Pseudo-second-order kinetic linear fitting of phen-TPA. c) Pseudo-first-order kinetic linear fitting of phen-TTA. d) Pseudo-second-order kinetic linear fitting of phen-TTA.


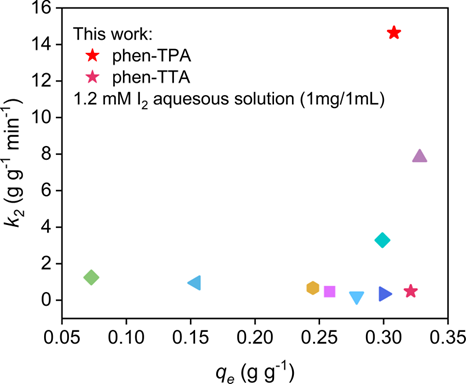


**Figure** **S15.** Comparison of the *q_e_* and *k_2_* with porous adsorbents for iodine adsorption. The porous adsorbents, whose iodine adsorption capacity were determined in 1.2 mM I_2_ aqueous solution with dosage of 1 mg mL^−1^, were collected for this comparison.


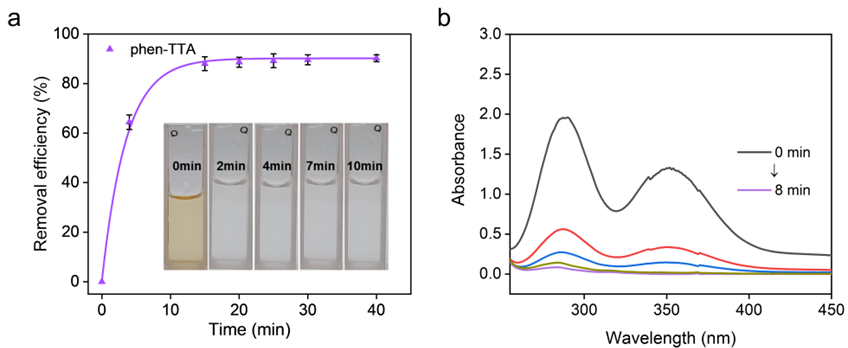


**Figure S16.** Adsorption of phen-TTA to iodine in 0.4 mM I_3_^−^ aqueous solution. a) Time dependent removal efficiency (inset: color changed of iodine solution during phen-TTA adsorption). b) Corresponding UV-Vis spectra.


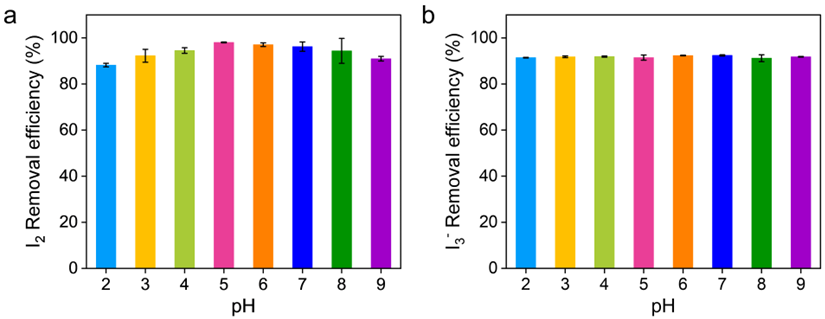


**Figure S17.** Removal of iodine pollutants from aqueous solution with broad pH range. a) 1.2 mM I_2_. b) 0.4 mM I_3_^−^.


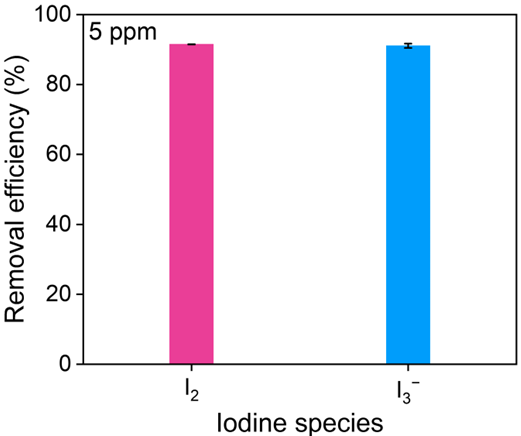


**Figure S18.** Removal of iodine pollutants from 5 ppm iodine-polluted water.


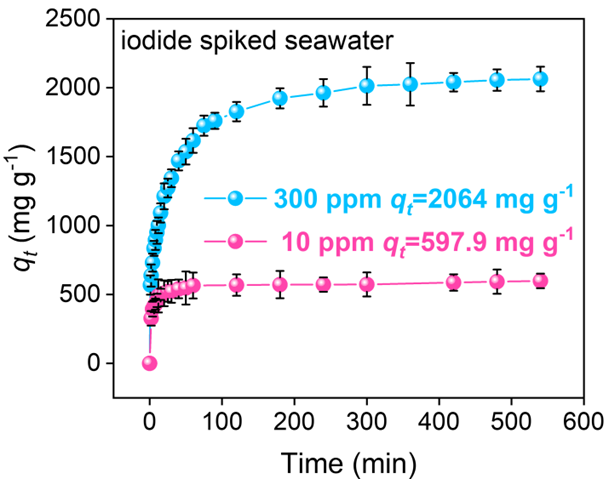


**Figure S19.** Time-dependent adsorption models of phen-TPA in iodine spiked (KI + I_2_) natural seawater.


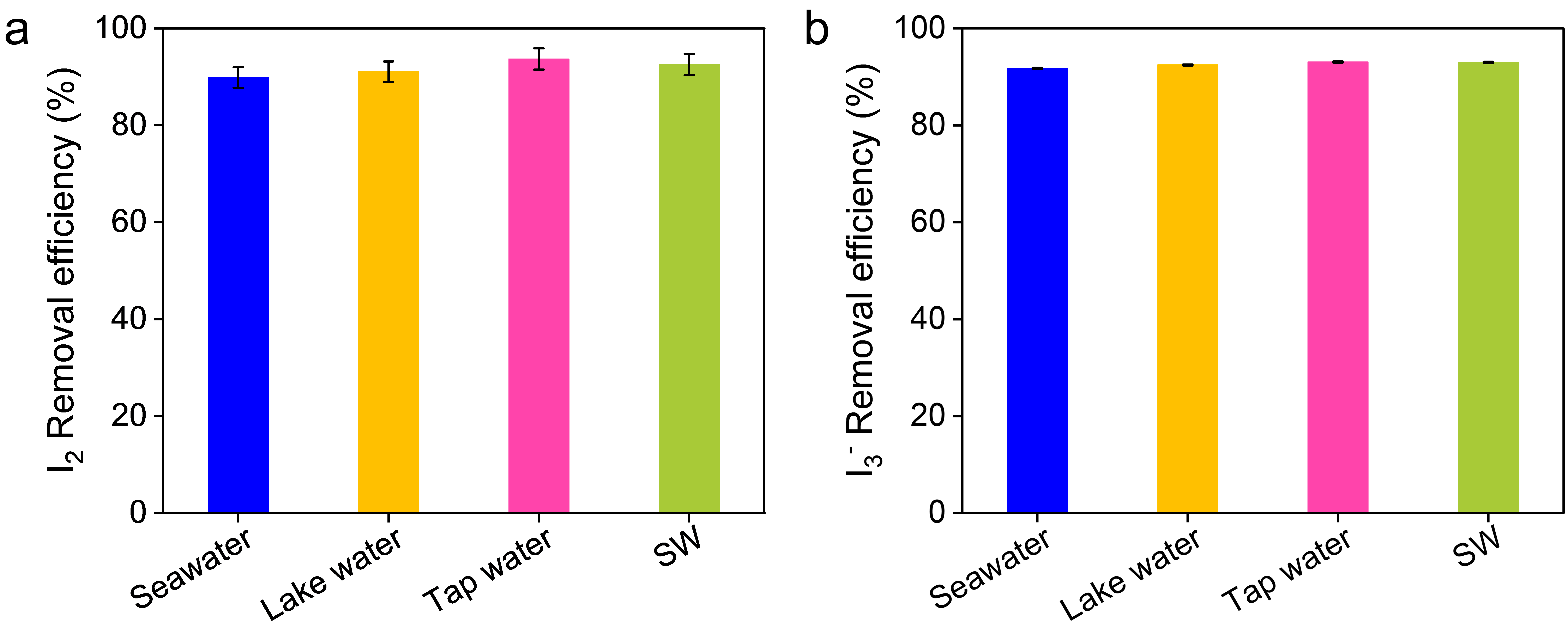


**Figure S20.** Removal of iodine pollutants from multiple iodine-polluted waters sources. a) 5 ppm I_2_. b) 5 ppm I_3_^−^.


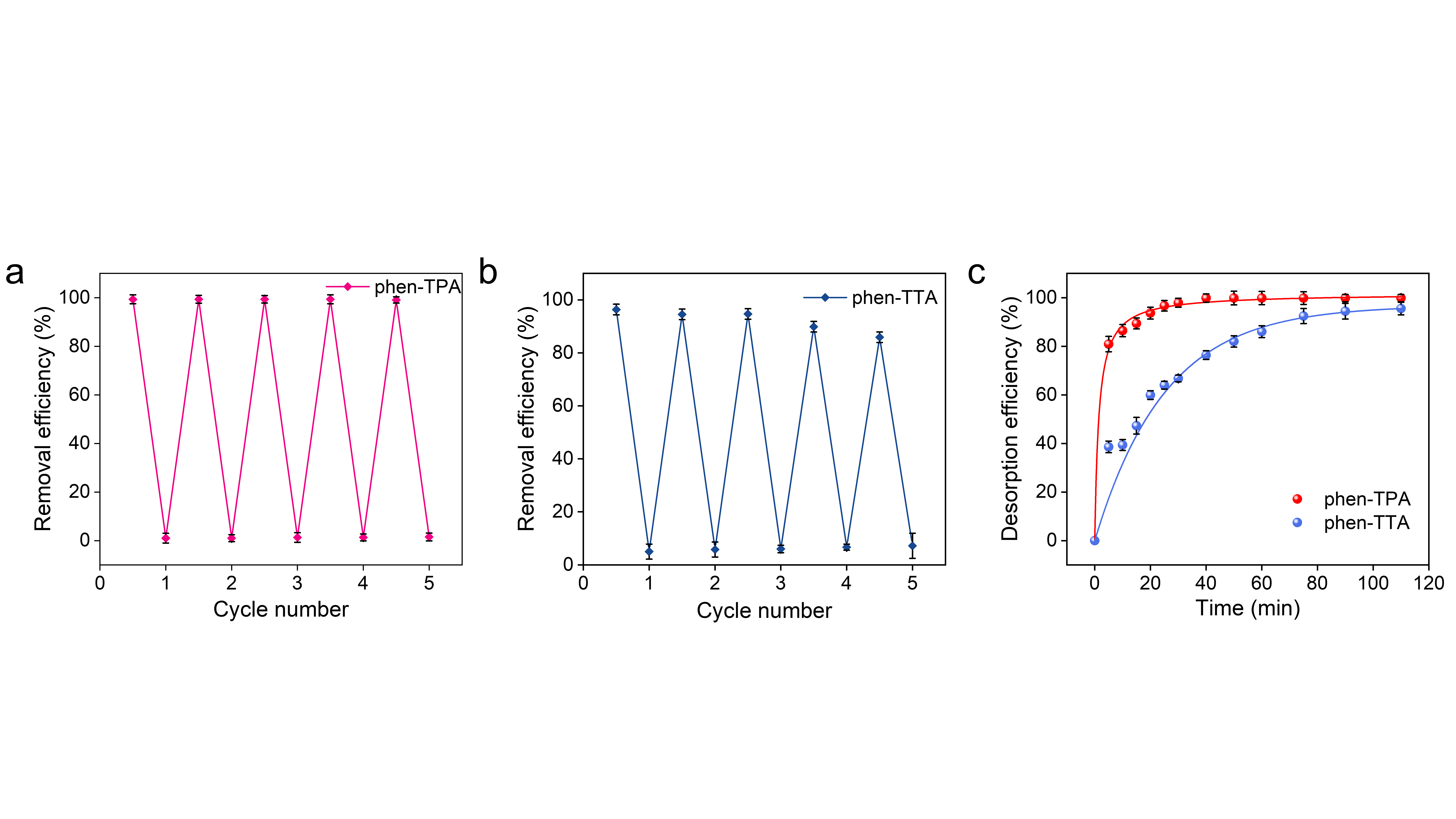


**Figure S21.** Recyclability of two COFs. a) Five cycles of adsorption and desorption of phen-TPA. b) Five cycles of adsorption and desorption of phen-TTA. c) Time-dependent desorption kinetics of phen-TPA and phen-TTA.


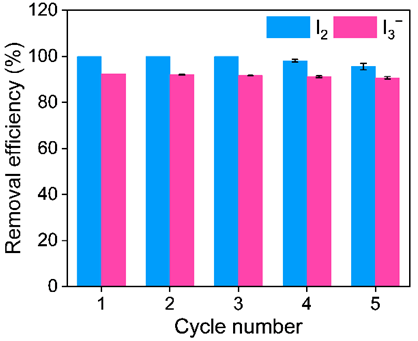


**Figure S22.** The iodine removal efficiency of recycled phen-TPA was evaluated using I_2_ aqueous solutions with 100 equivalent concentrations of competing anions (Cl^−^, Br^−^, NO_3_^−^, SO_4_^2−^) over five cycles.


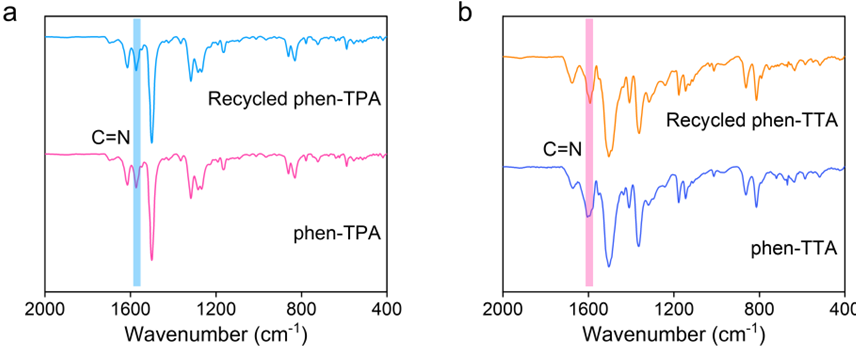


**Figure S23.** FT-IR spectra of recycled COFs. a) Recycled phen-TPA. b) Recycled phen-TTA.


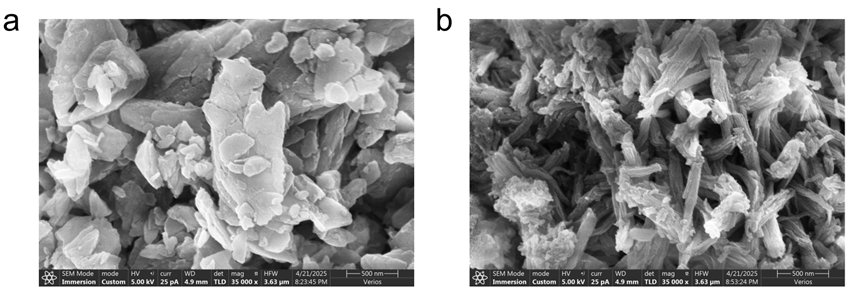


**Figure S24.** SEM images of recycled COFs. a) Recycled phen-TPA. b) Recycled phen-TTA.


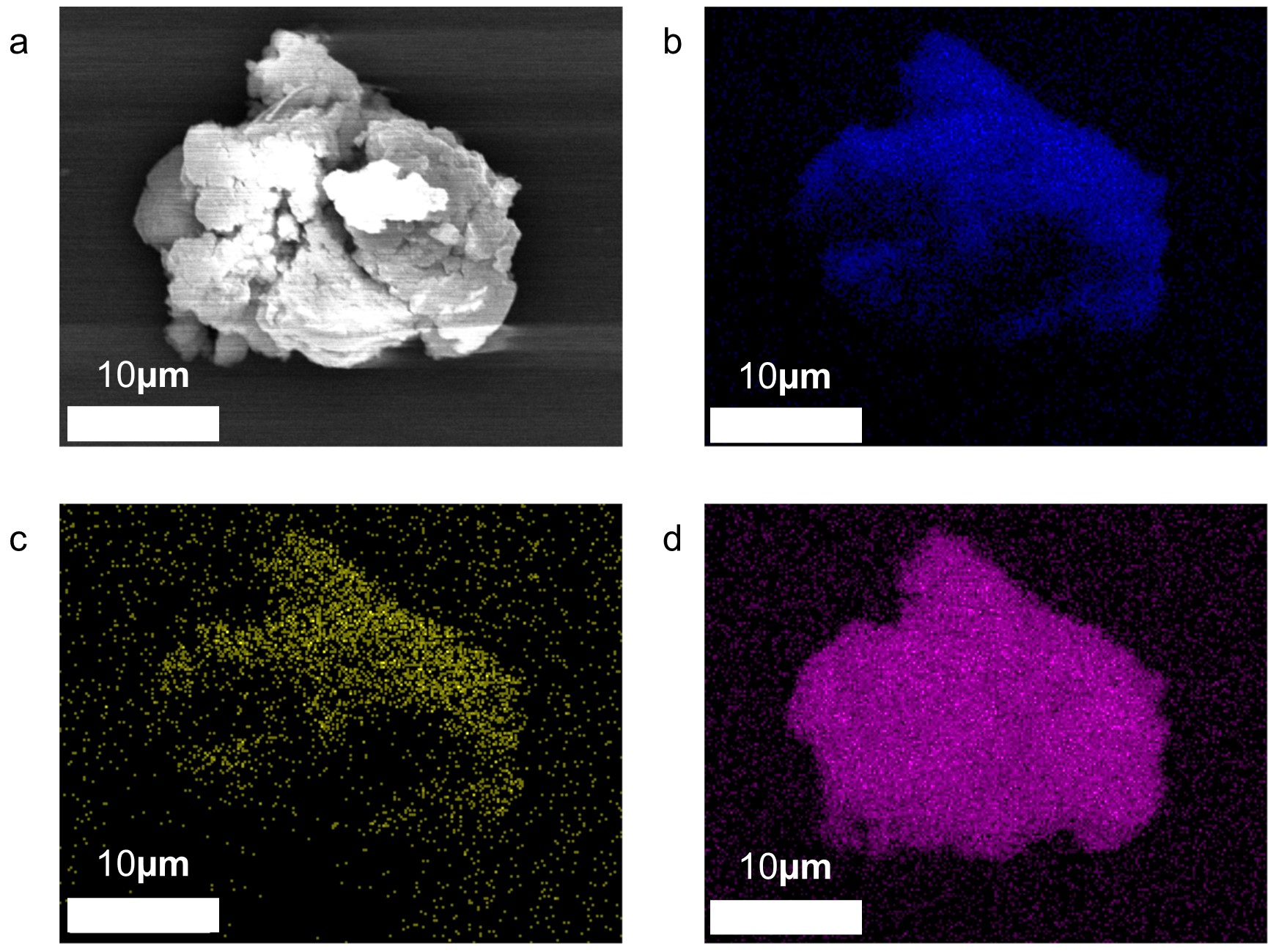


**Figure S25.** SEM and EDS mapping results of iodine-loaded phen-TTA. a) SEM image of I_2_@phen-TTA and SEM/EDS mapping for b) C. c) N. d) I.


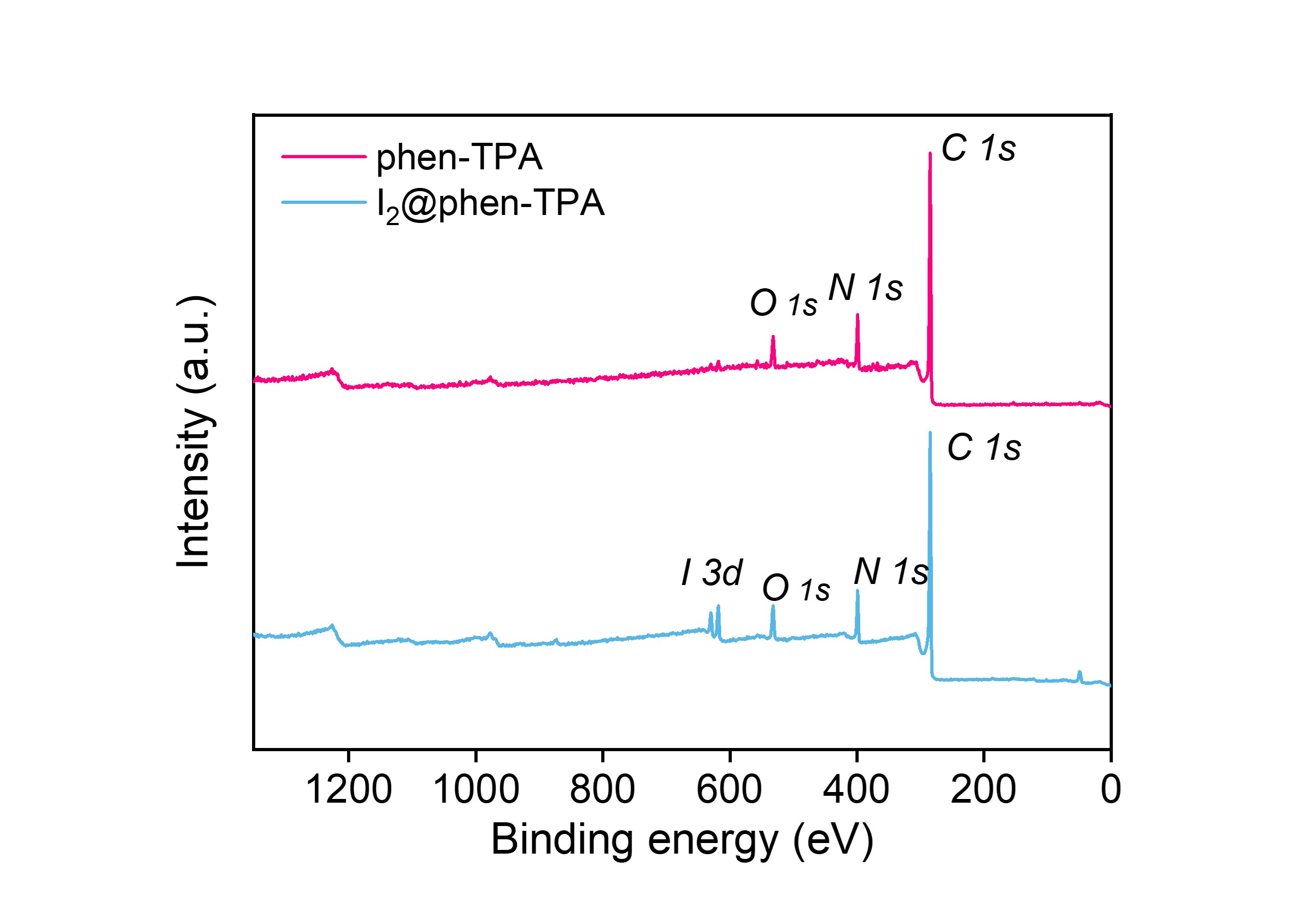


**Figure S26.** XPS full spectra of phen-TPA from 1.2 mM iodine solution before and after adsorption.


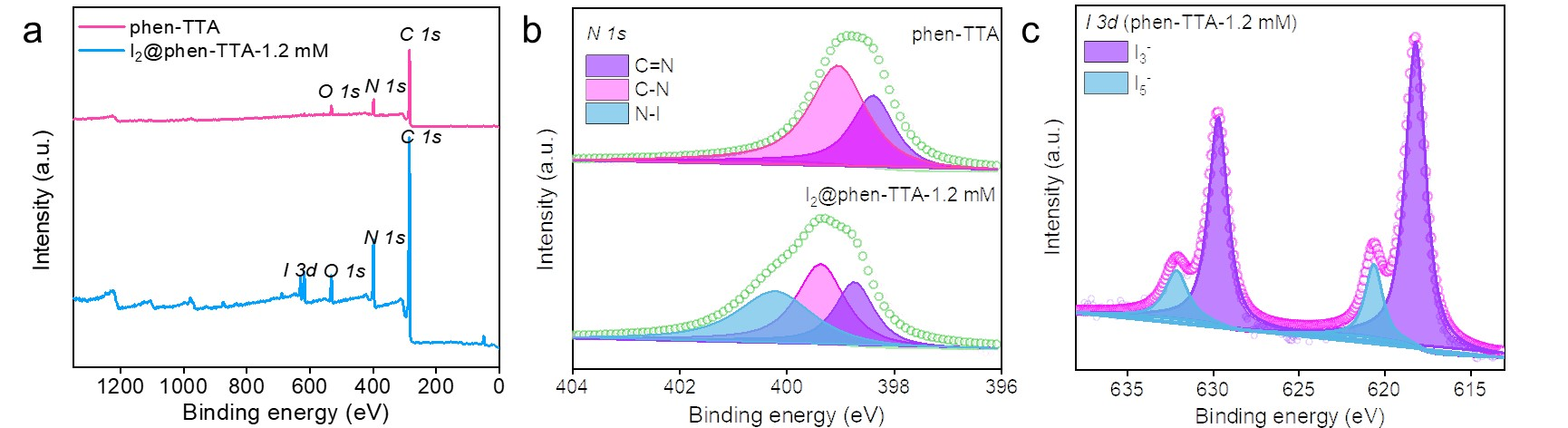


**Figure S27.** XPS spectra of iodine adsorbed by phen-TTA from 1.2 mM iodine solution. a) Full spectra. b) N *1s* spectra. c) I *3d* spectra.


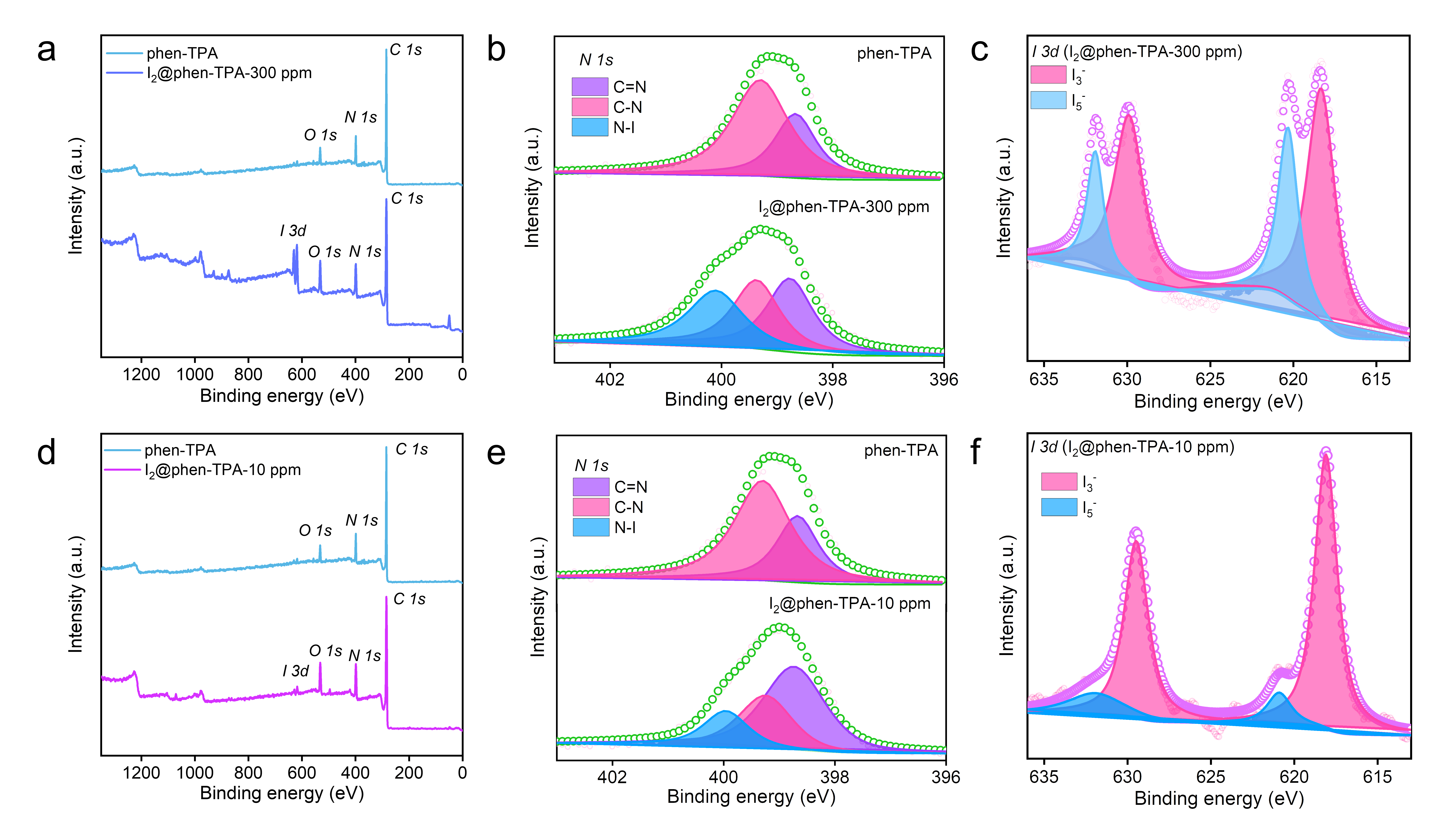


**Figure S28.** XPS spectra of iodine adsorbed by phen-TPA from real seawater spiked with iodine. a) d) Full spectra. b) e) N *1s* spectra. c) f) I *3d* spectra.


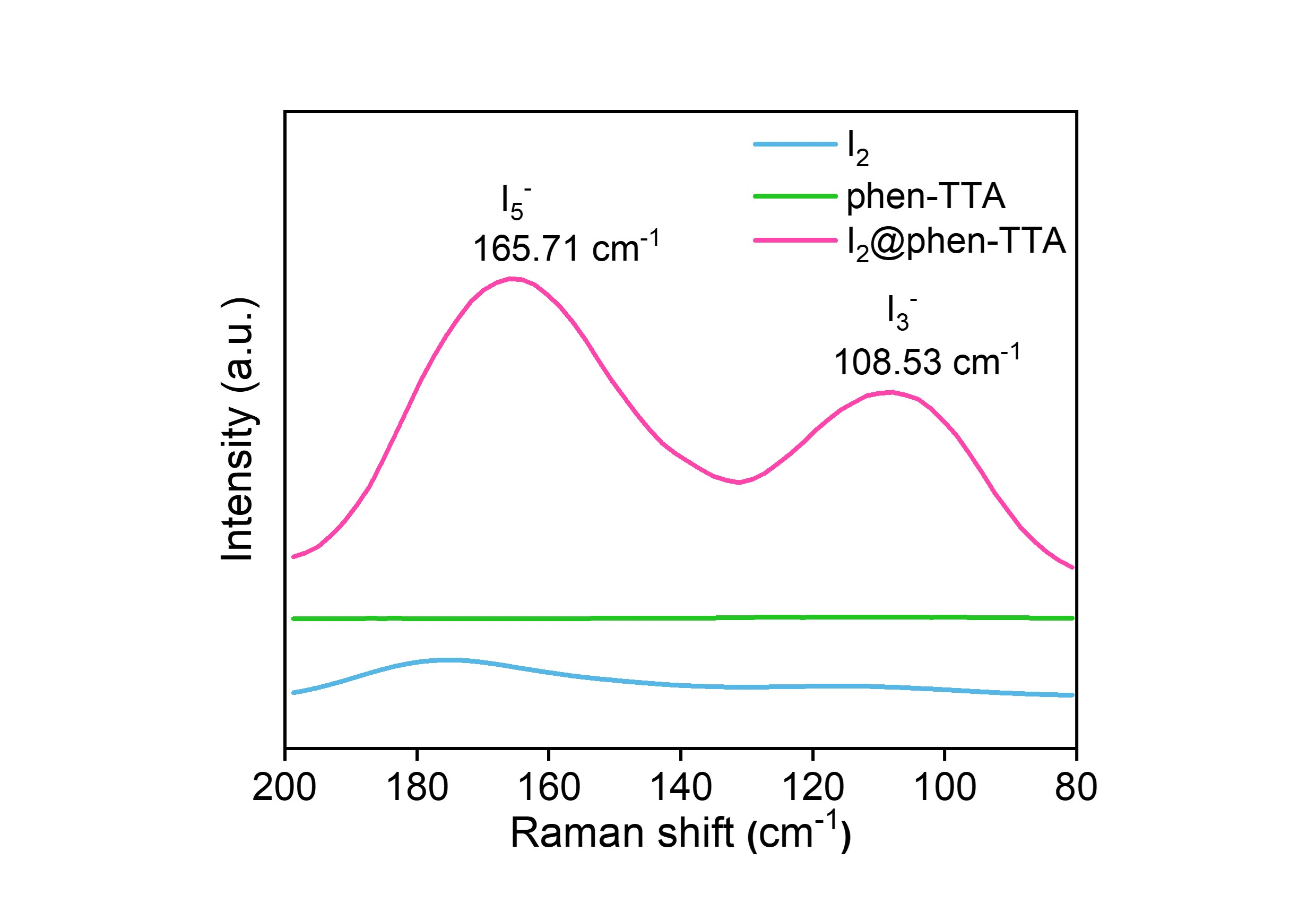


**Figure S29.** Raman spectra of pristine I_2_, phen-TTA and I_2_@phen-TTA.


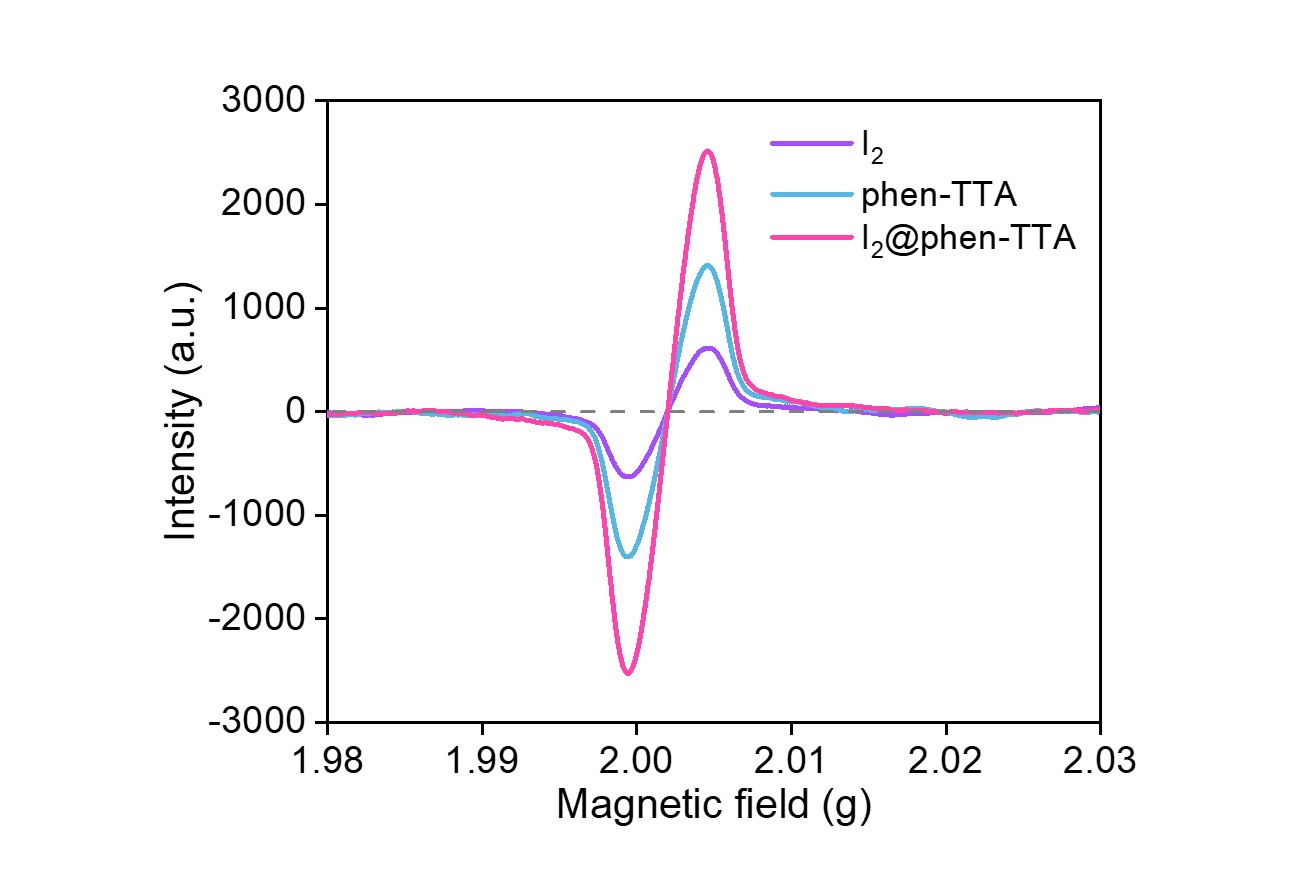


**Figure S30.** EPR spectra of pristine I_2_, phen-TTA and I_2_@phen-TTA.


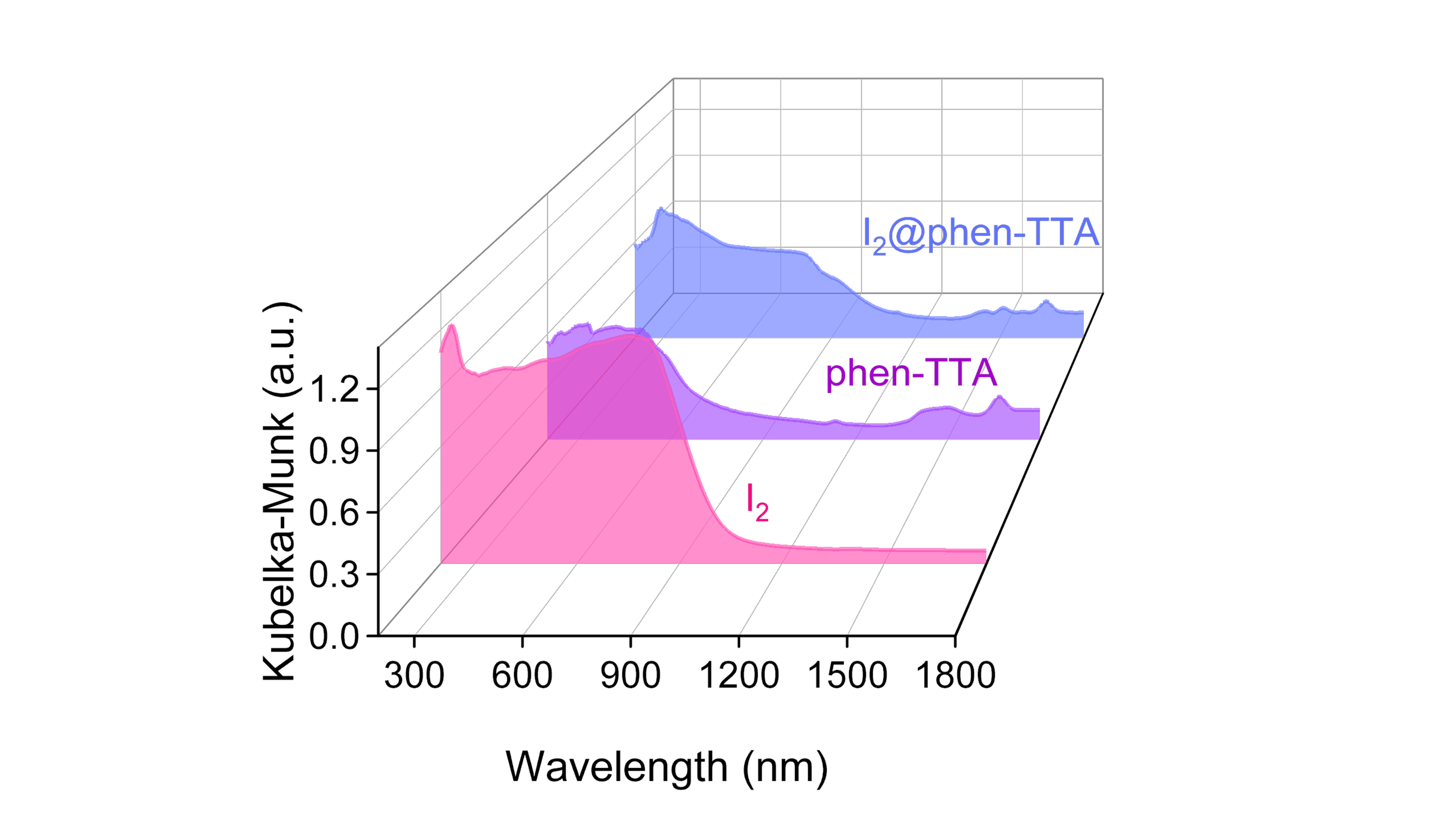


**Figure S31.** UV/Vis/NIR spectra of pristine I_2_, phen-TTA and I_2_@phen-TTA.


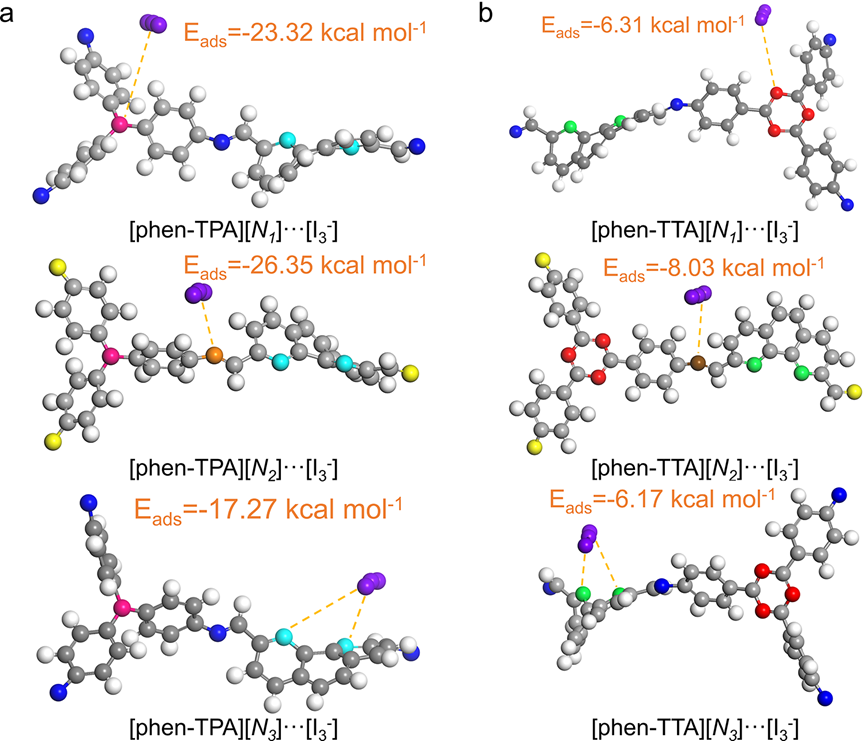


**Figure S32.** Binding energy calculation between I_3_^−^ and phen-TPA or phen-TTA. a) phen-TPA and b) phen-TTA.


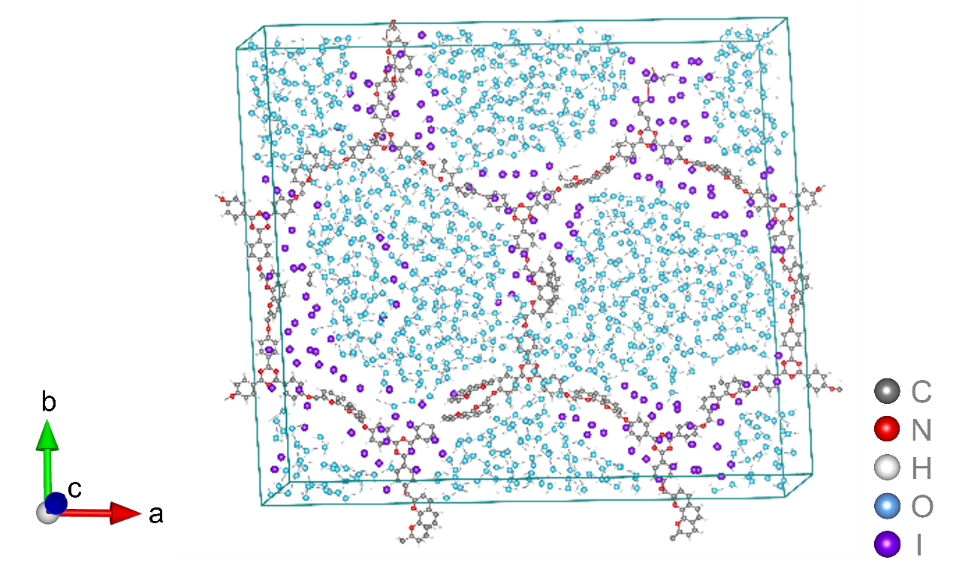


**Figure S33.** Molecular dynamics simulation of phen-TTA at 200 ps.


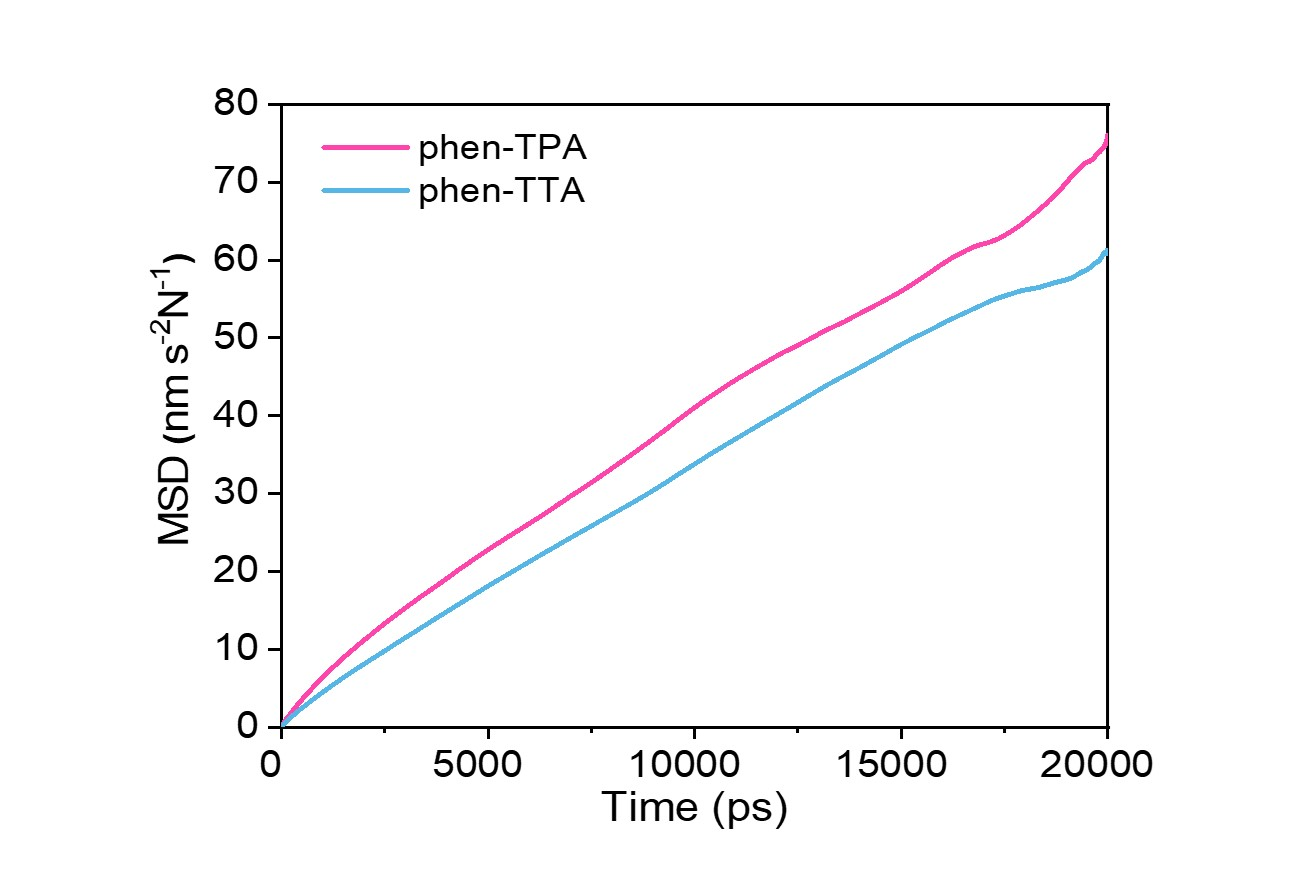


**Figure S34.** MSD curves of phen-TPA and phen-TTA.

**Table S1.** Iodine uptake capacities and adsorption kinetics constants of different adsorbent materials in iodine aqueous solution.

| Type | Materials | *k_2_* (g g^- 1^min^-1^) | Removal efficiency (%) | Reference |
| --- | --- | --- | --- | --- |
| COF | phen-TPA | 14.640 | 99.1 | **This work** |
| POP | C[4]P-HEPM | 10.220 | 97.6 | 14 |
| POP | C[4]P-TEPM | 8.547 | 95.0 | 14 |
| POP | C[4]P-BTP | 7.814 | 99.8 | 15 |
| POP | C[4]P-TPE | 3.286 | 92 | 15 |
| POP | Fc-PP-POP | 1.130 | 99.3 | 16 |
| POP | C[4]P-DPP | 0.951 | 44.4 | 15 |
| POP | C[4]P-BT | 0.668 | 71.1 | 15 |
| MOF | C-poly-1_20_ | 0.620 | 91.0 | 17 |
| COF | phen-TTA | 0.487 | 98.9 | **This work** |
| POP | C[4]P-TTP | 0.470 | 73.0 | 15 |
| MOF | C-poly-1_50_ | 0.390 | 42.0 | 17 |
| MOF | C-poly-1_80_ | 0.350 | 55.0 | 17 |
| MOF | C-poly-1_100_ | 0.340 | 42.0 | 17 |
| POP | C[4]P-BP | 0.218 | 71.1 | 15 |
| MOF | N-MOF-PAN fibers | 0.143 | 94.6 | 18 |
| Carbons | PTEP | 0.0142 | 77.0 | 19 |

**Note:** COF: covalent-organic framework; POP: porous organic polymers; MOF: metal organic framework.

**Table S2.** Comparison of adsorption capacity with different materials (Conditions: 1.2 mM I_2_, dosage: 1mg 1mL^−1^).

| Type | Materials | *q_e_* (g g^−1^) | *k_2_*  (g g^−1^ min^−1^) | Reference |
| --- | --- | --- | --- | --- |
| POP | C[4]P-BTP | 0.328 | 7.814 | 15 |
| COF | phen-TTA | 0.321 | 0.487 | **This work** |
| COF | phen-TPA | 0.308 | 14.640 | **This work** |
| POP | BPy-Box⋅4PF6 | 0.3 | 0.3404 | 20 |
| POP | C[4]P-TPE | 0.299 | 3.286 | 15 |
| POP | C[4]P-BP | 0.279 | 0.218 | 15 |
| POP | C[4]P-TTP | 0.258 | 0.470 | 15 |
| POP | C[4]P-BT | 0.245 | 0.668 | 15 |
| POP | C[4]P-DPP | 0.154 | 0.951 | 15 |
| Supramolecular Framework | Q[8]-(4-AP) | 0.0728 | 1.248 | 21 |

**Table S3.** Experiments on the adsorption capacity of different adsorbents for iodine in I_3_^−^ aqueous solutions.

| Type | Materials | Iodine uptake capacity (g g^-1^) | Reference |  |
| --- | --- | --- | --- | --- |
| COF | phen-TPA | 11.90 | **This work** |  |
| NAS | SUPE-py-Imine-Cage | 9.01 | 22 |  |
| COF | | phen-TTA | 8.90 | **This work** |
| NAS | SUPE-py-Amine-Cage | 8.10 | 22 |  |
| COF | COFA-1 | 7.13 | 23 |  |
| POP | PP-3 | 5.90 | 24 |  |
| POP | PP-2 | 5.80 | 24 |  |
| POP | PP-4 | 5.10 | 24 |  |
| COF | COFP-1 | 4.81 | 23 |  |
| POP | C[4]P-HEPM | 4.45 | 14 |  |
| POP | PP-1 | 4.20 | 24 |  |
| POP | C[4]P-TEPM | 4.02 | 14 |  |
| POP | PP-5 | 4.00 | 24 |  |
| MOF | MOF nanosheets | 3.70 | 25 |  |
| HOF | HcOF-4 | 3.57 | 26 |  |
| MOF | N-MOF-PAN fibers | 3.56 | 18 |  |
| POP | Compound 1 | 3.55 | 27 |  |
| POP | C[4]P-BTP | 3.24 | 15 |  |
| HOF | HcOF-2 | 3.23 | 26 |  |
| POP | CaCOP3 | 3.10 | 28 |  |
| POP | Compound 2 | 3.04 | 25 |  |
| HOF | HcOF-3 | 3.00 | 26 |  |
| POP | C[4]P-TPE | 2.99 | 15 |  |
| POP | CaCOP2 | 2.81 | 28 |  |
| POP | C[4]P-TTP | 2.51 | 15 |  |
| POP | CaCOP1 | 2.40 | 28 |  |
| POP | C[4]P-BP | 2.37 | 15 |  |
| POP | C[4]P-BT | 2.32 | 15 |  |
| POP | CalCOP1 | 2.32 | 29 |  |
| HOF | HcOF-1 | 2.10 | 30 |  |
| POP | CalCOP2 | 1.76 | 29 |  |
| POP | C[4]P-DPP | 1.58 | 15 |  |
| MOF | {[Mn_2_(oxdz)_2_(tpbn)(H_2_O)_2_]·2C_2_H_5_OH}_n_ | 1.10 | 31 |  |
| aluminum macrocycle-faced cages | AIMC-1 | 0.89 | 32 |  |
| POP | CalCOP3 | 0.35 | 29 |  |
| MOF | SCNU-Z4 | 0.33 | 33 |  |
| POP | CalCOP4 | 0.15 | 29 |  |

**Note:** NAS: nonporous amorphous sorbents; HOF: hydrogen-bonded cross-linked organic frameworks.

**Table S4.** phen-TPA and phen-TTA on Bader charge at *N_1_,* *N_2_* and *N_3_* sites.

| Materials | phen-TPA | | | | phen-TTA | | |
| --- | --- | --- | --- | --- | --- | --- | --- |
|  | *N_1_* | *N_2_* | *N_3_* | *N_1_* | | *N_2_* | *N_3_* |
| Bader charge | 0.98 | 2.01 | 1.82 | 0.06 | | 2.06 | 1.91 |

**Reference**

1. C. I. Pearce, E. A. Cordova, W. L. Garcia, S. A. Saslow, K. J. Cantrell, J. W. Morad, O. Qafoku, J. Matyas, A. E. Plymale, S. Chatterjee, J. Kang, F. C. Colon, T. G. Levitskaia, M. J. Rigali, J. E. Szecsody, S. M. Heald, M. Balasubramanian, S. Wang, D. T. Sun, W. L. Queen, R. Bontchev, R. C. Moore, V. L. Freedman, *Sci. Total Environ.* **2020**, 716, 136167.
2. J. Hutter, M. Iannuzzi, F. Schiffmann, J. VandeVondele, *Wiley Interdiscip. Rev. Comput. Mol. Sci.* **2014**, 4, 15.
3. B. G. Lippert, J. H. Parrinello, Michele, *Molecular. Physics.* **1997**, 92, 477.
4. J. VandeVondele, J. Hutter, *J. Chem. Phys.* **2007**, 127, 114105.
5. Goedecker, Teter, Hutter, *Phys. Rev. B, Condens. Matter* **1996**, 54, 1703.
6. S. Grimme, J. Antony, S. Ehrlich, H. Krieg, *J. Chem. Phys.* **2010**, 132, 19.
7. D. Van Der Spoel, E. Lindahl, B. Hess, G. Groenhof, A. E. Mark, H. J. C. Berendsen, *J. Comput. Chem.* **2005**, 26, 1701.
8. S. Páll, M. J. Abraham, C. Kutzner, B. Hess, E. Lindahl, presented at *Solving Software Challenges for Exascale*, Cham, **2015**.
9. M. J. Abraham, T. Murtola, R. Schulz, S. Páll, J. C. Smith, B. Hess, E. Lindahl, *SoftwareX* **2015**, 1-2, 19.
10. H. J. C. Berendsen, D. van der Spoel, R. van Drunen, *Comput. Phys. Commun.* **1995**, 91, 43.
11. L. Martinez, R. Andrade, E. G. Birgin, J. M. Martinez, *J. Comput. Chem.* **2009**, 30, 2157.
12. H. J. C. Berendsen, J. R. Grigera, T. P. Straatsma, *J. Phys. Chem.* **1987**, 91, 6269.
13. . Wang, R. M. Wolf, J. W. Caldwell, P. A. Kollman, D. A. Case, *J. Comput. Chem.* *2004*, **25**, 1157.
14. Z. Y. Zheng, Q. Y. Lin, L. H. Xie, X. L. Chen, H. Zhou, K. H. Lin, D. S. Zhang, X. D. Chi, J. L. Sessler, H. Y. Wang, *J. Mater. Chem. A.* **2023**, 11, 13399.
15. L. H. Xie, Z. Y. Zheng, Q. Y. Lin, H. Zhou, X. F. Ji, J. L. Sessler, H. Y. Wang, *Angew. Chem. Int. Ed.* **2022**, 61, e202113724.
16. C. Miao, L. Chu, D. Guo, X. Ding, W. Guo, S. Wang, J. Sheng, J. Zhang, Z. Wang, B. Zhou, *J. Environ. Chem. Eng.* **2023**, 11, 110514.
17. X. H. Xu, Y. X. Li, L. Zhou, N. Liu, Z. Q. Wu, *Chem. Sci.* **2022**, 13, 1111.
18. D. Y. Chen, T. T. Ma, X. Y. Zhao, X. F. Jing, R. Zhao, G. S. Zhu, *Acs Appl. Mater. Inter.* **2022**, 14, 47126.
19. Y. C. Yin, Y. Yang, G. J. Liu, H. L. Chen, D. Gong, Y. M. Ying, J. R. Fan, S. J. Liu, Z. Li, C. H. Wang, Z. Y. Guo, Z. K. Li, C. B. Yu, G. F. Zeng, *Chem. Eng. J.* **2022**, 441, 11.
20. B. Wu, Z. W. Li, F. Lin, R. Tang, W. Zhang, H. Liu, G. Ouyang, Y. Tan, *J. Hazard. Mater.* **2024**, 465, 133449.
21. Y. Lu, Z. C. Yu, T. T. Zhang, D. W. Pan, J. J. Dai, Q. Li, Z. Tao, X. Xiao, *Small* **2023**, DOI: 10.1002/smll.20230817510.
22. W. Zhou, A. Li, M. Zhou, Y. Xu, Y. Zhang, Q. He, *Nat. Commun.* **2023**, 14, 5388.
23. X. F. Li, Z. M. Jia, J. Zhang, Y. D. Zou, B. Jiang, Y. D. Zhang, K. W. Shu, N. Liu, Y. Li, L. J. Ma, *Chem. Mat.* **2022**, 34, 11062.
24. M. Avais, S. Chattopadhyay, *J. Mater. Chem. A.* **2022**, 10, 20090.
25. C. X. Yu, X. J. Li, J. S. Zong, D. J. You, A. P. Liang, Y. L. Zhou, X. Q. Li, L. L. Liu, *Inorg. Chem*. **2022**, 61, 13883.
26. X. F. Jiang, X. Z. Cui, A. J. E. Duncan, L. Li, R. P. Hughes, R. J. Staples, E. V. Alexandrov, D. M. Proserpio, Y. Y. Wu, C. F. Ke, *J. Am. Chem. Soc.* **2019**, 141, 10915.
27. A. Sen, S. Sharma, S. Dutta, M. M. Shirolkar, G. K. Dam, S. Let, S. K. Ghosh, *Acs Appl. Mater. Inter.* **2021**, 13, 34188.
28. D. An, L. Li, Z. Z. Zhang, A. M. Asiri, K. A. Alamry, X.-H. Zhang, *Mater.* *Chem. Phys.* **2020**, 239, 6.
29. Z. Z. Zhang, L. Li, D. An, H. X. Li, X. H. Zhang, *J. Mater. Sci.* **2020**, 55, 1854.
30. Y. X. Lin, X. F. Jiang, S. T. Kim, S. B. Alahakoon, X. S. Hou, Z. Y. Zhang, C. M. Thompson, R. A. Smaldone, C. F. Ke, *J. Am. Chem. Soc.* **2017**, 139, 7172.
31. A. Gogia, P. Das, S. K. Mandal, *Acs Appl. Mater. Inter.* **2020**, 12, 46107.
32. Y. J. Liu, Y. F. Sun, S. H. Shen, S. T. Wang, Z. H. Liu, W. H. Fang, D. S. Wright, J. Zhang, *Nat. Commun.* **2022**, 13, 10.
33. G. Q. Wang, J. F. Huang, X. F. Huang, S. Q. Deng, S. R. Zheng, S. L. Cai, J. Fan, W. G. Zhang, *Inorg. Chem. Front.* **2021**, 8, 1083.
